# An electron-density point-cloud framework for robust protein-ligand interaction prediction

**DOI:** 10.1038/s41467-026-74196-5

**Published:** 2026-06-11

**Authors:** Yujian Liu, Yutong Wang, Qingquan Wang, Meitang Peng, Yuan Chen, Yuechuan Lin, Dongxu Shen, Xiaoli Liu, Shidang Xu, Bin Liu

**Affiliations:** 1 School of Biomedical Sciences and Engineering, South China University of Technology, Guangzhou International Campus, Guangzhou, PR China; 2 Thrust of Artificial Intelligence, Information Hub, Hong Kong University of Science and Technology (Guangzhou), Guangzhou, PR China; 3 AiShiWeiLai AI Research, Beijing, PR China; 4 National Engineering Research Center for Tissue Restoration and Reconstruction, South China University of Technology, Guangzhou, PR China; 5 Guangdong Province Key Laboratory of Biomedical Engineering, South China University of Technology, Guangzhou, PR China; 6 Key Laboratory of Biomedical Materials of the Ministry of Education, South China University of Technology, Guangzhou, PR China; 7 Department of Chemical and Biomolecular Engineering, National University of Singapore, 4 Engineering Drive 4, Singapore, Singapore

**Keywords:** Computational biology and bioinformatics, Drug screening

## Abstract

Accurate protein-ligand affinity prediction typically depends on precise 3D coordinates, limiting robustness when structures are low-resolution or predicted. We introduce E-CloudBind, a framework that fuses electron-density point clouds with intrinsic molecular graphs to model non-covalent and covalent interactions without relying on sub-ångström accuracy. Ligand electron densities are obtained by semi-empirical quantum calculations, whereas protein pockets are represented by van der Waals-guided Gaussian point clouds, a physically motivated proxy that preserves interaction geometry while tolerating coordinate noise. Point-cloud encoders capture local non-covalent patterns and a heterogeneous graph neural network integrates them with covalent features for affinity regression. Across PDBbind splits and out-of-distribution scenarios, E-CloudBind matches or exceeds leading sequence-, graph- and structure-based baselines, with markedly reduced sensitivity to resolution and to experimental-versus-predicted proteins. Case studies further illustrate atom-level interpretability and large-scale virtual screening. By decoupling interaction learning from exact coordinates, E-CloudBind enables robust structure-based modeling on heterogeneous conditions.

## Abstract (web summary)

E-CloudBind improves drug-protein interaction (DPI) prediction by combining electron cloud representations with molecular graphs, enabling robust performance across low-resolution, predicted, and out-of-distribution structures for drug discovery.

## Introduction

Drug-protein interaction (DPI) prediction is central to modern drug discovery^1–3^. Although in vitro assays provide reliable affinity measurements, they are too slow and costly to explore the vast, discontinuous chemical space at scale^4–6^. Even high-throughput screening, which tests tens of thousands of compounds against a single target, is ultimately limited by reagent consumption and instrument time^7,8^. Deep learning surrogates have therefore emerged as an attractive alternative^9–16^. Early models rely on one-dimensional (protein sequences, SMILES)^10,13,14^ or two-dimensional (molecular graphs, contact maps)^11,15,16^ representation, which afford rich training data and architectural flexibility. Yet binding is governed by three-dimensional (3D) physicochemical complementarity; flattening either partner sacrifices geometric cues that are essential for recognizing shape, electrostatics and solvation, which in turn limits generalization to unseen proteins and chemotypes^13,17,18^.

**Fig. 1:**
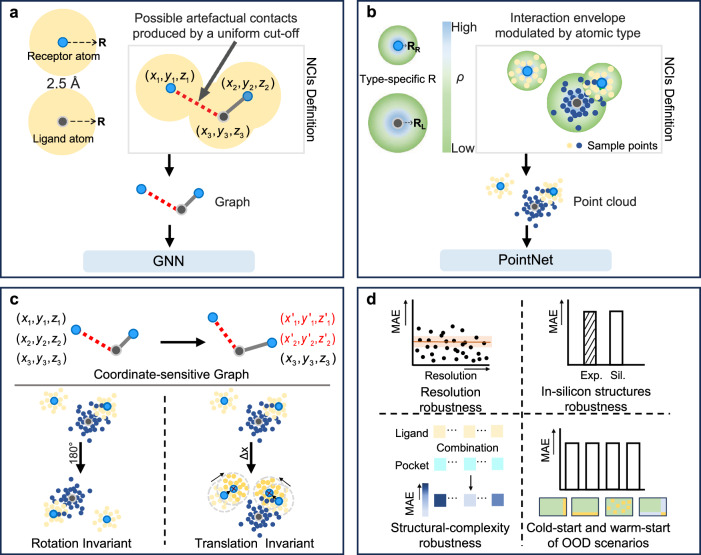
Electron-density-adaptive definition of receptor-ligand non-covalent interactions (NCIs) and a noise-tolerant feature pipeline. **a** Conventional drug-protein interaction (DPI) model prediction pipeline. Conventional DPI models adopt a uniform cut-off scheme, defining a NCI whenever any receptor and ligand atoms fall within a global Euclidean cutoff (e.g., 2.5 Å, yellow envelopes). Since the same radius is applied to all elements, atom identity is ignored, leading to spurious contacts in some cases (red dashed line). The constructed interaction graph is then passed into a graph neural network (GNN) for affinity prediction via message passing. **b** E-CloudBind prediction pipeline. E-CloudBind employs a density-adaptive scheme. Each atom is represented as a Gaussian electron cloud, and an interaction is recorded when its isosurfaces overlap. Type-specific radii (e.g., 1.2 Å for N, 1.7 Å for S) emerge naturally from local electron densities, preserving chemical identity and eliminating artefactual overlaps. The resulting electron cloud is encoded as a point cloud and input into a point-cloud encoder (PointNet) for affinity prediction. **c** Robustness of the point-cloud encoding. Conventional graph-based DPI models exhibit high sensitivity to coordinate uncertainty (top): even modest perturbations (Δx ≈ 0.5 Å) can reshuffle atomic contacts, leading to large fluctuations in features learned by the 3D GNN and increased prediction error. In contrast, E-CloudBind employs Gaussian sampling within adaptive envelopes (bottom) to generate a point-cloud representation that is inherently rotation- and translation-invariant, preserving the true contact geometry even under positional perturbations. **d** Performance and generalization. Density-adaptive envelopes and point-cloud model enable a physically grounded, resolution-robust description of NCIs, leading to improved affinity prediction. The approach excels not only on high-quality structural data, but also demonstrates strong robustness on low-resolution, experimental carbon-based (Exp.) and silicon-based (Sil.) structures, and four types of out-of-distribution (OOD) targets. Performance was evaluated using mean absolute error (MAE) and the Pearson correlation coefficient (Pearson).

Structure-aware networks^12,18,19^ promise a remedy, but their practical adoption has been slowed by the uneven landscape of available protein conformations^18,20–22^. Only a minority of proteins in today’s pharmacopeia have atomic-resolution (<2 Å) crystal or cryo-EM structures, and even those can harbor coordinate errors from crystal packing, radiation damage or model bias. Predicted models from algorithms such as AlphaFold^23,24^ have filled many gaps, yet their accuracy varies, particularly in flexible loops, low-confidence regions and side-chain orientations^25–28^. Conventional pipelines further amplify these issues by using fixed Euclidean distance cut-offs to define non-covalent contacts: small coordinate noise can spuriously create or erase interactions, corrupting the learning signal and degrading affinity prediction (Fig. 1)^3,12,17,18,20,22,29,30^. These limitations point to two requirements for robust DPI modeling: (1) encodings that inject physically grounded priors^31–33^ like reasoning over electron-density overlap or anisotropic atomic potentials rather than hard distance thresholds, and (2) resilience to structural heterogeneity across experimental resolutions and silicon-based predictions.

**Fig. 2:**
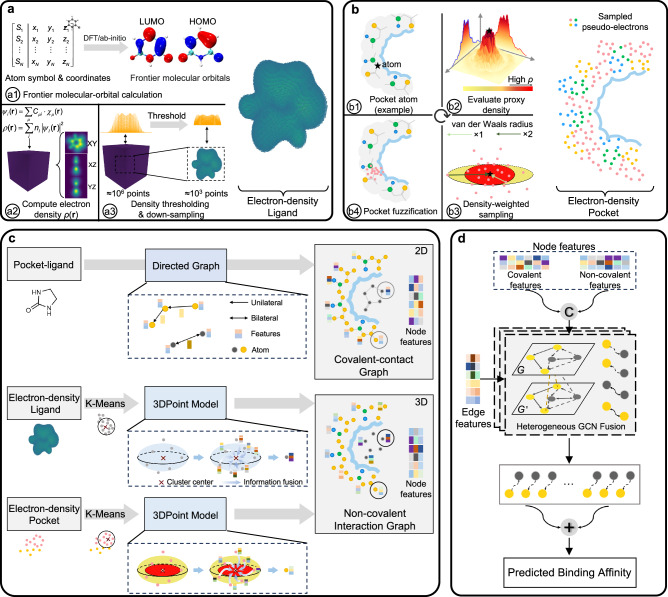
Workflow of E-CloudBind framework for binding-affinity prediction. **a** Quantum electron density modeling for ligand. Starting from atomic symbols and Cartesian coordinates, frontier molecular orbitals are computed using a quantum chemistry solver based on density functional theory or ab initio methods (DFT/ab-initio) (a1). While highest occupied/lowest unoccupied molecular orbital (HOMO/LUMO) are shown for visualization, downstream modeling uses the total electron density reconstructed from all occupied orbitals^70^. The resulting coefficients are used to evaluate the electron density ρ (a2), which is then thresholded and down-sampled to convert ≈10^6^ grid points into an ≈10^3^ point “electron-density ligand” cloud (a3). **b** Gaussian electron density modeling for pocket. For each pocket atom (b1), a multivariate Gaussian kernel centered at the atom position provides a proxy for the local electron distribution (b2). The kernel width is linked to the van der Waals (vdW) radius, and density-weighted sampling generates pseudo-electrons with a higher concentration inside the 1 $\times$ r_vdW_ region than in the 1-2 $\times$ r_vdW_ shell (b3). Repeating the procedure for all atoms yields an “electron-density pocket” that captures non-covalent interaction geometry while remaining tolerant to coordinate noise (b4). **c** Covalent graph construction and non-covalent interaction extraction. Atomic covalent connectivity is represented as a directed chemical graph and embedded by a covalent graph encoder (top). The electron-density ligand and pocket are individually clustered by K-means clustering, and each cluster is processed through a 3D point-cloud network to extract non-covalent embeddings (middle, bottom). The curved blue arrow denotes the direction of information aggregation among the point clouds. **d** Feature fusion and prediction. Covalent and non-covalent node and edge feature vectors are concatenated and fed into a heterogeneous graph convolutional network (GCN) that jointly reasons over both interaction types. The resulting edge-level scores are aggregated to deliver the predicted binding affinity. By combining quantum-mechanical density information with topology-aware encoders, E-CloudBind captures type-specific interaction ranges, preserves anisotropic electrostatics, and remains robust across experimental resolutions and AlphaFold2-predicted structures^23^.

Here we introduce E-CloudBind, a framework that represents complexes as electron-density point clouds rather than ball-and-stick coordinates (Fig. 2). Grounded in quantum-chemical principles of electron-density overlap and dispersion^34^, E-CloudBind replaces hard distance cutoffs with Gaussian electron clouds whose type-specific spreads are physically derived (Fig. 1b). Two atoms are considered in contact when their isosurfaces intersect, yielding a chemically faithful, resolution-agnostic interaction graph (Fig. 2a, b). A point-cloud encoder then aggregates local cloud clusters, capturing higher-order hydrogen bonding, π-stacking and van der Waals complementarity without demanding sub-ångström coordinate accuracy. Because the model operates on densely sampled point clouds, it is inherently insensitive to small coordinate perturbations (Fig. 1c), remaining robust to spatial deviations arising from conformational heterogeneity and preserving reliable inference of non-covalent interactions. Benchmarks on crystal (0.75-3.5 Å) and AlphaFold structures show that this physically grounded approach delivers state-of-the-art affinity prediction and maintains robust performance over a wide spectrum of structural quality.

## Results

### E-CloudBind framework

E-CloudBind is a regression model that predicts the binding affinity between proteins and ligands by generating the electron-cloud structures of proteins and ligands and their graphs. Fig. 2 illustrates the architecture of E-CloudBind, where in the structures of proteins and ligands (Fig. 2a, b) is fundamental to E-CloudBind’s insensitivity to structural quality and its incorporation of non-covalent interaction information. Specifically, we replace the conventional 3D ball-and-stick model with a physically grounded electron-cloud representation. Unlike classical approximations, this model captures the intrinsic electromagnetic basis of intermolecular forces such as hydrogen bonds, van der Waals forces, and π-π stacking. By modeling them as dipole-dipole interactions, the model enhances generalization through a more faithful representation of interaction physics^35–37^. Furthermore, conventional structure-based DPI methods^12,17,20,22,30^ often depend on 3D ball-and-stick structure and fixed Euclidean thresholds (e.g., 5 Å) to determine non-covalent interactions, rendering them highly sensitive to structural inconsistencies arising from differences in resolution, prediction algorithms, or experimental conditions (Fig. 1a, c). Based on the aforementioned reason, we employ electron-cloud structure to model non-covalent interactions more realistically. For proteins, we introduce a van der Waals radius-based sampling strategy that selectively perturbs protein structures, reducing complexity while preserving essential chemical information, as computing full electron-cloud densities is both unnecessary and computationally intensive due to the localized nature of interaction pockets and the large atomic scale (Fig. 2b).

To fully harness atomic and multi-bond force information, E-CloudBind is divided into three modules: a non-covalent interaction point-cloud encoder, a covalent interaction heterogeneous graph encoder, and a multi-bond fusion module. Initially, the electron-cloud structures of proteins/ligands are clustered into groups via K-means clustering, with each cluster representing the non-covalent interaction region an atom can influence, which are then input into the point-cloud encoder to extract non-covalent interaction information (Fig. 2c). Subsequently, the inherent covalent bonds within the molecular graph, where vertices represent atoms and edges represent types of covalent bonds, are input into the message-passing encoder to extract covalent interaction information. After obtaining both covalent and non-covalent information of the atoms, E-CloudBind integrates them as the node attributes of the protein-ligand heterogeneous graph (Fig. 2d). Through the heterogeneous graph encoder, it fuses information from different types of edges and nodes to achieve feature integration. Lastly, after representation learning with E-CloudBind, the heterogeneous graph encoder's output is fed into the task layer to predict protein-ligand interactions.

### Robustness analysis across heterogeneous structural quality

**Fig. 3:**
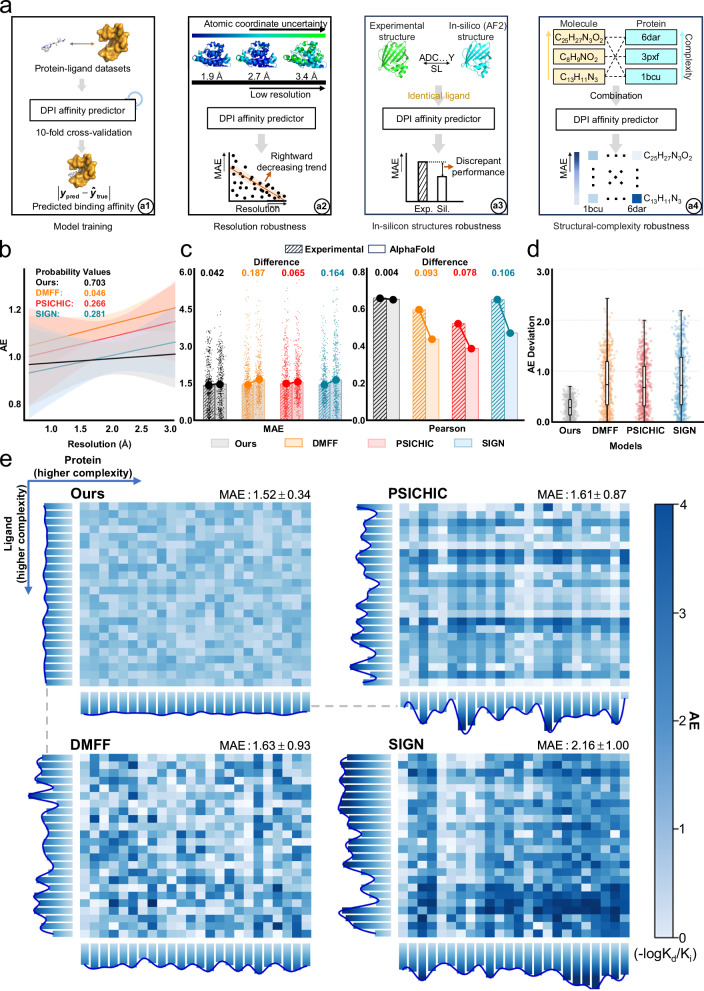
Robustness and generalization of the DPI model in comparison with three state-of-the-art baselines (PSICHIC^20^, SIGN^17^ and DMFF^46^). **a** Experimental design. a1 10-fold cross-validation was performed on the union of PDBbind 2013, 2016 and 2019^42^. a2 To quantify robustness to crystallographic resolution, model performance was evaluated on experimentally resolved structures across different crystallographic resolutions (Å). a3 Generalization to silico-predicted structures (Sil.) was assessed by replacing experimental structures (Exp.) with AlphaFold2^23^ (AF2) models for identical amino-acid sequences. a4 Structural-complexity robustness was evaluated by combining ligands of varying molecular complexity with proteins of differing fold complexity and measuring performance across the resulting grid. **b** Robustness of resolution on model performance. Absolute error (AE) increases with resolution (Å) across four methods. Solid lines represent least-squares fits, and shaded areas indicate 95% confidence intervals. The slopes for E-CloudBind, SIGN, PSICHIC, and DMFF increase progressively, with DMFF exhibiting a statistically significant slope (*p* = 0.046), indicating high sensitivity to resolution. In contrast, the stable slope of E-CloudBind suggests robust performance across varying resolutions. **c** Structural robustness across experimental and AF2 predictions. For every model, paired bars report mean absolute error (MAE, left) and Pearson correlation coefficient (Pearson, right). E-CloudBind attains both the lowest MAE and the highest Pearson on experimental as well as AF2 structures, with a deviation of only 0.012 MAE units between sources. **d**,**e** E-CloudBind consistently outperforms baselines in complexity-based evaluations. In Fig. d, the distribution of per-pair AE deviations from the corresponding method’s MAE on the DAVIS dataset^47^ indicates that E-CloudBind exhibits a lower and more stable median deviation (0.280) than all baselines. Fig. e visualizes per-pair AE as heat maps across methods, with columns ordered by increasing protein structural complexity (Relative Contact Order, RCO)^48^ and rows by increasing ligand structural complexity (Bertz Complexity Index, BertzCT)^49^. Each cell represents a protein-ligand pair, and lighter colors denote lower AE. E-CloudBind achieves the lowest MAE (1.520) and standard deviation (0.340) across all combinations, whereas the baseline models degrade in specific complexity regimes and generalize less consistently.

With the advent of protein structure prediction methods such as AlphaFold, a vast number of proteins that previously lacked structural information due to experimental limitations can now be modeled with silicon-based approaches to yield 3D conformations^38–41^. However, notable discrepancies remain between carbon-based experimentally resolved structures and silicon-based predicted models, particularly in terms of resolution, local conformational accuracy, and binding states. This raises a critical challenge: how can we robustly and effectively predict protein-ligand binding affinities using 3D structures of widely varying quality? To address the issue, we adopt a strategy that integrates electron-cloud representations and molecular graphs to simulate atomic interactions upon binding. A 10-fold cross-validation scheme is employed to predict the binding affinity score for each protein-ligand pair in the PDBbind datasets^42,43^ (Fig. 3a1 and Supplementary Fig. 1), with training configurations detailed in Supplementary Table 1. Specifically, we compared with state-of-the-art methods for different types of inputs and achieved superior performance, as detailed in Supplementary Table 4. E-CloudBind achieved the lowest mean absolute error (MAE, 1.059), which is 0.038, 0.120, and 0.079 lower than the three baseline models SIGN, DMFF, and PSICHIC, respectively. It also achieved the highest Pearson correlation coefficient (Pearson, 0.667), exceeding SIGN, DMFF, and PSICHIC by 0.013, 0.060, and 0.049, respectively. In addition to the above baselines that span different input modalities, we benchmarked E-CloudBind against three recent methods, EHIGN^30^, Boltz-2^44^, and FlowDock^45^, using the same 10-fold cross-validation protocol. In these comparisons, E-CloudBind consistently achieved the lowest MAE and the highest Pearson. Descriptions of all the above methods and the corresponding results are provided in the Supplementary Information, specifically in the Benchmarking Against State-of-the-Art Methods section, Supplementary Table 4 and Supplementary Fig. 1.

To begin with, the quality of protein structures, particularly their resolution, has long been recognized as a critical factor influencing the predictive accuracy of 3D structure-based DPI models^20^. Structures with lower resolution introduce substantial uncertainty in atomic coordinates, especially in flexible side chains, active binding pockets, and solvent-exposed regions. Such uncertainties adversely affect the extraction of geometric and spatial features, thereby impairing the model’s ability to identify non-covalent interactions and estimate binding affinities with precision (Fig. 3a2). To assess this effect, the influence of structural resolution on prediction outcomes was systematically investigated (Fig. 3b). The proposed structure-based model, E-CloudBind, was evaluated in comparison with three representative models employing different input modalities: structure-based DMFF^46^, sequence-based PSICHIC^20^, and graph-based SIGN^17^. All baseline models demonstrated a consistent increase in prediction error as structural resolution declined (i.e., higher Å values), with regression slopes of 0.065, 0.060, and 0.053, respectively. The absolute error-resolution relationship was quantified using ordinary least squares linear regression, and the significance of the linear trend was assessed with a parametric F-test, with the reported p-value corresponding to a standard t-test on the slope coefficient and evaluating whether the slope differs from zero. In this setting, a larger p-value indicates that no statistically significant linear trend is observed between absolute error (AE) and resolution. Compared with representative methods spanning three input modalities (structure, graph, and sequence), E-CloudBind exhibited lower AE and greater stability, with a regression slope of only 0.017. Consistently, the fitted regression curve and the high p-value (0.703), which is higher than the p-values of the three baselines, further support E-CloudBind’s improved robustness to resolution variations.

Next, we investigated the impact of protein structure sources, experimental carbon-based methods versus silicon-based predictions from AlphaFold2^23^, on model performance. To assess this, each ligand-target pair was re-evaluated by substituting the protein structure with its corresponding AlphaFold2 prediction, allowing quantification of performance shifts (Fig. 3a3). As illustrated in Fig. 3c, although PSICHIC relies primarily on sequence inputs and is not directly dependent on structures, its transformation into molecular graphs involves AlphaFold2-predicted geometries. Similar to SIGN, which infers non-covalent interactions based on Euclidean distance between atoms, PSICHIC exhibited increased sensitivity to atomic displacement, resulting in an MAE increase of 0.065 and a decline of 0.078 in Pearson correlation coefficient. DMFF, which directly utilizes 3D coordinates, experienced a more substantial performance drop with predicted structures, with MAE increasing by 0.187 and Pearson correlation coefficient decreasing by 0.093. In contrast, E-CloudBind, despite also being structure-based, showed strong structural generalization, with only a modest increase in MAE of 0.042 and a negligible reduction in Pearson correlation coefficient of 0.004.

Finally, to further evaluate generalization under out-of-distribution (OOD) scenarios involving considerable structural variability, an OOD benchmark was constructed based on the DAVIS dataset^47^. Protein-ligand pairs were generated through combinatorial partitioning guided by protein and ligand structural complexity, resulting in 576 test samples (Fig. 3a4). Figure 3d visualizes the distribution of per-pair absolute error (AE) deviations relative to the corresponding method’s MAE. E-CloudBind attains the lowest median deviation (0.280) and the smallest dispersion, with medians 0.456, 0.397, and 0.442 lower than DMFF, PSICHIC, and SIGN, respectively. Figure 3e reports complexity-conditioned AE heat maps for the methods, with columns ordered by increasing Relative Contact Order (RCO)^48^ and rows ordered by increasing Bertz Complexity Index (BertzCT)^49^, where each cell corresponds to one protein-ligand pair and lighter colors indicate lower AE. In these stratified comparisons, PSICHIC tends to perform better in regimes with moderate ligand complexity, SIGN shows relatively lower error in simpler protein regimes, and DMFF exhibits less consistent behavior with larger AE variability. In contrast, E-CloudBind maintains stable and accurate performance across the full range of protein and ligand complexity, supporting strong generalization under structurally challenging OOD settings.

### Ablation and generalization analysis

**Fig. 4:**
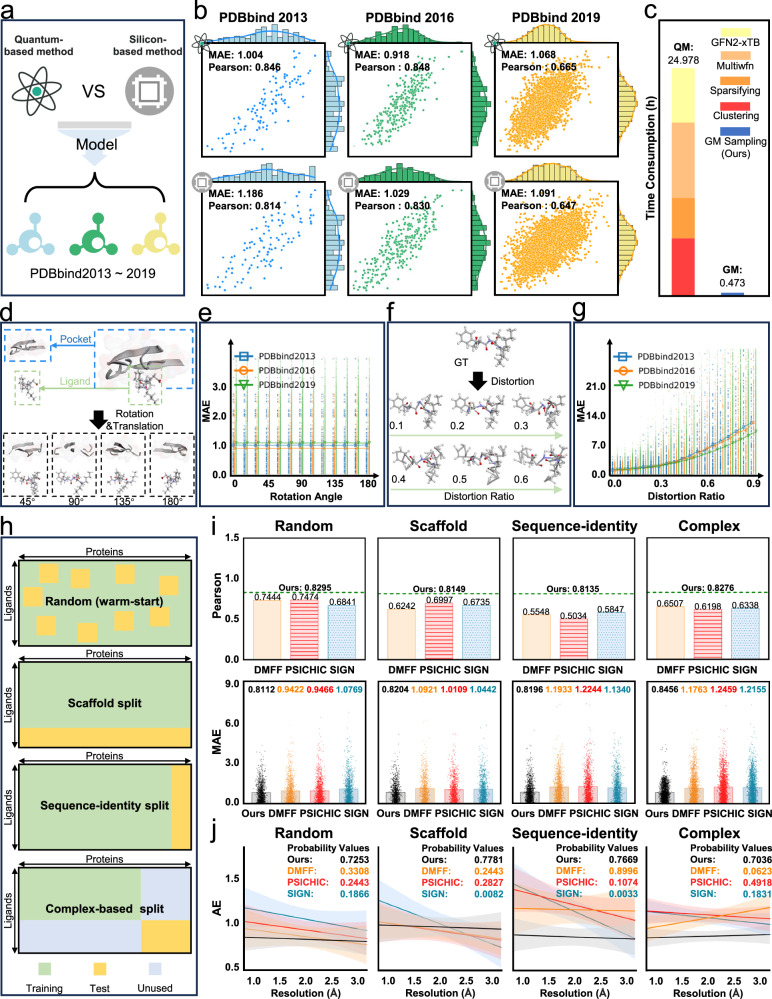
Efficiency of van der Waals-guided Gaussian sampling strategy and generalization of the DPI model. **a** Computational alternatives for modeling ligand electron clouds. Electron-density clouds obtained from xTB quantum calculations (left) and the proposed van der Waals Gaussian sampler (right) are each coupled to the same model and tested on PDBbind 2013-2019^42^. **b** Performance and prediction distribution on PDBbind test sets (blue/green/yellow)for quantum-derived electron-density modeling (top) and Gaussian-based modeling (bottom). Differences are within ≤ 0.032 for mean absolute error (MAE) and ≤ 0.020 for Pearson correlation coefficient (Pearson), indicating that the inexpensive Gaussian procedure achieves comparable performance to quantum-derived modeling. **c** Computational cost of electron-cloud modeling. Runtime comparison of the quantum calculation pipeline (QM) versus the proposed van der Waals Gaussian sampling pipeline (GM) on the PDBbind dataset, showing fewer preprocessing steps and lower preprocessing time for GM. **d**,**e** Rigid transformation invariance. The ligand and pocket electron clouds were jointly rotated around the z-axis by 0° to 180° at 15° intervals, with a fixed translation applied. Performance remains nearly unchanged across rotations, with only minor deviations from the 0° setting. **f**,**g** Electron-cloud distortions under non-rigid transformations. Ligand edges and the corresponding electron-cloud clusters were progressively corrupted to mimic non-rigid strain. Performance degrades monotonically from 0 to 90% corruption, indicating reliance on preserving correct geometric patterns in the electron cloud. **h** Data-partition strategies. Warm-start performance was evaluated under a random split, whereas cold-start performance was assessed under holdout splits based on (i) ligand scaffolds, (ii) protein sequence-identity clusters, and (iii) joint protein-ligand complex similarity for evaluation on previously unseen patterns. **i** Comparative performance under warm-start and cold-start splits. E-CloudBind yields the highest Pearson and lowest MAE in every setting. Across the three cold-start regimes, its MAE increases by only 0.026, whereas SIGN^17^, PSICHIC^20^ and DMFF^46^deteriorate by 0.101-0.235. **j** Resolution sensitivity under warm-start and cold-start splits. Absolute error (AE) versus crystallographic resolution for the four data splits (solid line = least-squares fit; shaded band = 95% confidence). E-CloudBind retains a near-zero slope in all splits, while baseline models show marked error inflation as resolution deteriorates.

To assess the contribution of the proposed van der Waals-inspired electron-like cloud structural strategy, a comparative experiment was designed to evaluate the effect of two structural methods on model performance (Fig. 4a): (1) electron density approximation based on calculations, and (2) multivariate Gaussian sampling regulated by parameterized van der Waals radii (Supplementary Fig. 2, 3). Under the condition that all protein-ligand pairs remained fixed and other external factors were held constant, ligands were processed using both approaches and evaluated across three benchmark datasets: PDBbind2013, PDBbind2016, and PDBbind2019. Figure 4b presents the predicted-versus-true scatter plots for the two methods on all datasets, along with corresponding MAE, Pearson correlation coefficient, and distribution patterns of the data points. The resulting performance differences between the two structural methods were minimal, and their data distributions were highly similar. Notably, the Gaussian sampling method achieved significantly higher computational efficiency than the semi-empirical GFN2-xTB approach. These results suggest that the proposed electron-like cloud structural method, parameterized by van der Waals radii, serves as a computationally efficient and performance-consistent alternative for structural representation in affinity prediction models. In Fig. 4c, we further quantify the computational cost associated with the two preprocessing pipelines. For the quantum-derived electron-density workflow, GFN2-xTB (6.7.0) is used to obtain the wavefunction information, Multiwfn reconstructs the three-dimensional density grid, and sparsification followed by clustering is applied to produce the electron-cloud representation used by E-CloudBind. Over the full PDBbind collection of 19,545 ligands, the GFN2-xTB and Multiwfn steps required 5.9 h and 8.2 h, respectively, whereas sparsification and clustering required 4.4 h and 6.4 h, respectively. By contrast, the proposed Gaussian-based modeling requires only a one-time offline procedure in which multivariate Gaussian sampling is performed for each atom based on its van der Waals radius, reducing the total preprocessing time to 0.5 h. The training time and inference speed of E-CloudBind are reported in Supplementary Table 3.

To quantify the sensitivity of E-CloudBind to variations in electron cloud, we conducted two evaluations that distinguish geometry-preserving rigid transformations from geometry-altering non-rigid distortions. For rigid transformations (Fig. 4d), we jointly rotated the ligand and pocket electron-cloud point sets around the z-axis by 0°, 15°, 30°, …, 180° and applied a fixed translation, and observed nearly unchanged performance across rotations, with only minor deviations from the 0° baseline (MAE (0.00408 ± 0.00240) and Pearson correlation coefficient (0.00094 ± 0.00092), Fig. 4e). For non-rigid distortions, we constructed corrupted inputs by first perturbing a fraction $r$ of ligand edges to emulate non-rigid strain and identify affected ligand atoms. We then applied geometric corruption only within the electron-cloud clusters associated with these atoms by replacing a fraction $r$ of point coordinates with random positions sampled from a locally expanded region (Fig. 4f). As $r$ increased from 0% to 90%, performance degraded monotonically, with higher MAE and lower Pearson correlation (Fig. 4g). Mild corruption can still preserve some informative signal due to the structural properties of the point cloud, whereas higher corruption levels make predictions unreliable, indicating that substantial geometric disruption prevents the extraction of reliable binding-related cues. Together, these results indicate that E-CloudBind is insensitive to rigid-coordinate changes but relies on preserving the correct geometric patterns in the electron cloud.

In addition, the previous section demonstrated that E-CloudBind exhibits strong, robust performance across out-of-distribution structural conditions with varying levels of similarity. To further examine its generalization capabilities under shifts in training data distribution, two evaluation paradigms were employed: transductive (warm-start) and inductive (cold-start) settings. Specifically, the transductive setting involved random partitioning of the dataset into training and testing subsets. The inductive setting introduced a greater distributional divergence by holding out training and testing splits based on (i) protein sequence similarity, (ii) ligand scaffolds, and (iii) joint protein-ligand complex similarity^50^, ensuring evaluation on previously unseen patterns (Fig. 4h). As shown in Fig. 4i, E-CloudBind consistently achieved superior generalization performance across both settings, yielding the lowest MAE (0.811) and highest Pearson correlation coefficient (0.830), with minimal error fluctuations of 0.021 and 0.008, respectively. In contrast, baseline models exhibited clear performance degradation under the inductive condition, marked by a notable increase in MAE and a decline in Pearson. Furthermore, Fig. 4j illustrates that E-CloudBind maintained stable prediction accuracy across protein structures with varying resolution, regardless of transductive or inductive partitioning. No significant performance drops were observed due to structural perturbations or partition-induced shifts. In comparison, models such as SIGN, PSICHIC, and DMFF demonstrated a clear trend of increasing prediction error as structural resolution declined, reinforcing the broader applicability and robustness of E-CloudBind in practical deployment scenarios. Taken together, these results indicate that (1) Gaussian-based electron density modeling serves as a significantly faster and more cost-effective alternative to quantum-derived density calculations, with negligible loss in predictive accuracy; and (2) the explicit non-covalent encoding and spatial representation adopted in E-CloudBind endow the model with strong generalization capabilities across diverse ligand scaffolds, protein families, and structural resolutions.

### Case analysis and large-scale screening

**Fig. 5:**
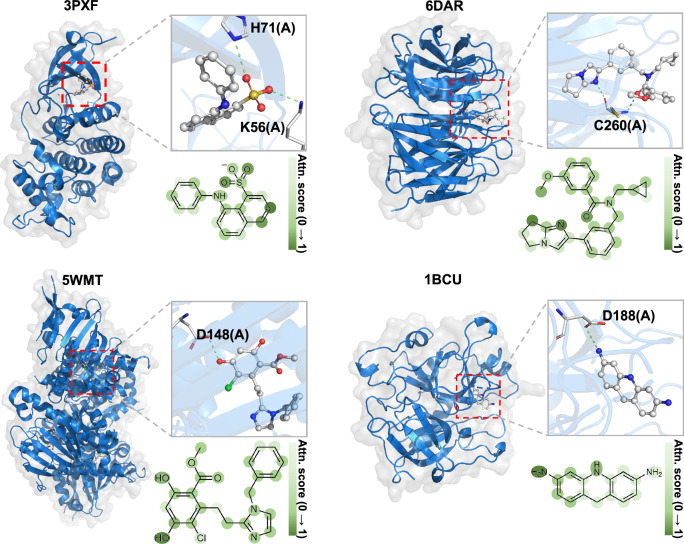
Consistent highlighting of interaction sites in diverse complexes. To visualize model interpretability in identifying non-covalent interactions (specifically hydrogen bonds), four representative protein-ligand complexes spanning a range of protein structural complexity were selected and ordered from low to high by Relative Contact Order (RCO) (PDB IDs: 3PXF, 6DAR, 5WMT, and 1BCU)^48^. Regardless of the complexity level of the protein or ligand, the model consistently assigned higher attention weights to non-carbon atoms on the ligands that were likely to form non-covalent interactions within the protein binding pocket.

To further evaluate the interpretability of the proposed model in recognizing non-covalent interactions, a progressive structural complexity strategy was adopted. Four representative protein-ligand complexes were selected to span a range of protein structural complexity quantified by Relative Contact Order (RCO), ordered from low to high as 3PXF^51^, 6DAR^52^, 5WMT^53^, and 1BCU^54^. Their non-covalent interactions, such as hydrogen bonding, were examined by visualization-based analysis. Figure 5 presents the results of this analysis, with additional target-specific outcomes provided in Supplementary Fig. 4. Despite the differences in the structural complexity of the selected proteins and ligands, the model exhibited a consistent attention pattern across all cases. In particular, non-carbon atoms on the ligand, especially those associated with polar functional groups located in the potential binding pocket, were assigned higher attention weights. This result indicates that the model is capable of effectively capturing key interaction regions, thereby confirming its interpretability in the context of non-covalent interaction identification.

**Fig. 6:**
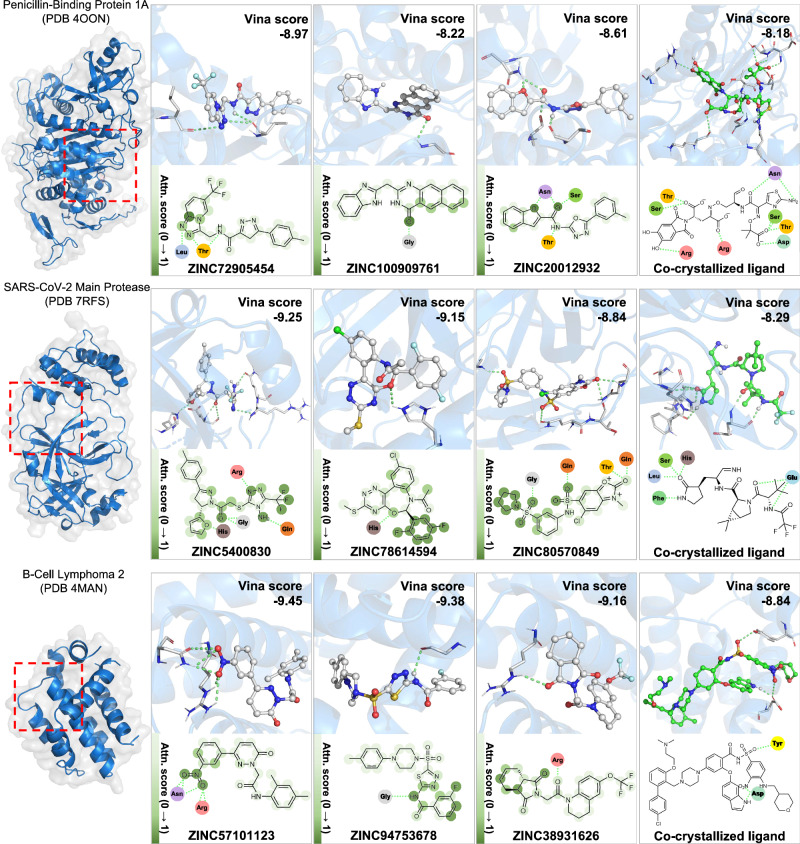
Prospective virtual screening guided by the E-CloudBind affinity predictor. Three therapeutically relevant targets were selected: Penicillin-Binding Protein 1 A (PBP1A; PDB: 4OON), the SARS-CoV-2 Main Protease (Mpro; PDB: 7RFS) and the B-Cell Lymphoma 2 (BCL-2; PDB: 4MAN). A library of 80,383 drug-like compounds from ZINC^55^ was evaluated by DPI and the top 5% of candidates for each protein were re-docked with AutoDock Vina^80^. For every target the left-most column shows the crystal structure with the binding pocket highlighted (red dashed box). The next three columns depict representative high-ranking candidates. Upper panels display the best-scoring Vina pose, annotated with the docking score. Lower panels visualize residue- and atom-level attention weights inferred by E-CloudBind; darker blue denotes higher contribution. The right-most column provides the co-crystallized ligand for reference, docked and analyzed in the same fashion. Across all systems, the attention hotspots coincide with hydrogen-bond donors/acceptors and π-stacking residues identified in the crystal complexes, confirming that E-CloudBind learns chemically meaningful non-covalent patterns. Notably, many screened compounds exhibit more favorable Vina score values (e.g., −9.45 for BCL-2) than the co-crystallized ligands (−8.84), highlighting chemotypes worthy of experimental follow-up. The combination of rapid Gaussian-based electron density modeling, accurate affinity prediction and interpretable attention maps demonstrates E-CloudBind’s suitability for structure-based lead discovery.

To further assess the model’s practical utility, three well-established drug targets were selected: Penicillin-Binding Protein 1 A (PBP1A; PDB: 4OON), SARS-CoV-2 Main Protease (Mpro; PDB: 7RFS), and B-Cell Lymphoma 2 (BCL-2; PDB: 4MAN). A total of 80,383 drug-like molecules were retrieved from the ZINC database for virtual screening^55^. The top 5% of candidates, ranked by the model’s predicted affinity scores, were subjected to molecular docking for validation. Specifically, RDKit^56^ was used to generate 3D conformations of the selected compounds, followed by molecular docking using AutoDock Vina to determine their optimal binding poses. Figure 6 displays the Vina scores and binding conformations of three top-ranking molecules docked to the 4OON, 7RFS and 4MAN targets, along with the atom-level attention scores predicted by the model. We used AutoDock Vina to determine the binding affinity of the candidate molecule (lower Vina scores indicate better binding). For comparison, we used the co-crystallized ligand for docking and compared its Vina score with the Vina score of the candidate molecule. The comparison results showed that the candidate molecule has a more stable binding posture than the co-crystallized ligand. Notably, a high degree of consistency was observed between the model’s attention distributions and the key polar interaction sites revealed by docking, thereby further supporting the model’s accuracy and interpretability in identifying drug-target interactions. Comparison with docking-derived binding modes indicates that the model’s attention mechanism reliably highlights critical non-covalent interaction regions, underscoring its potential applicability in virtual screening and rational drug design.

**Fig. 7:**
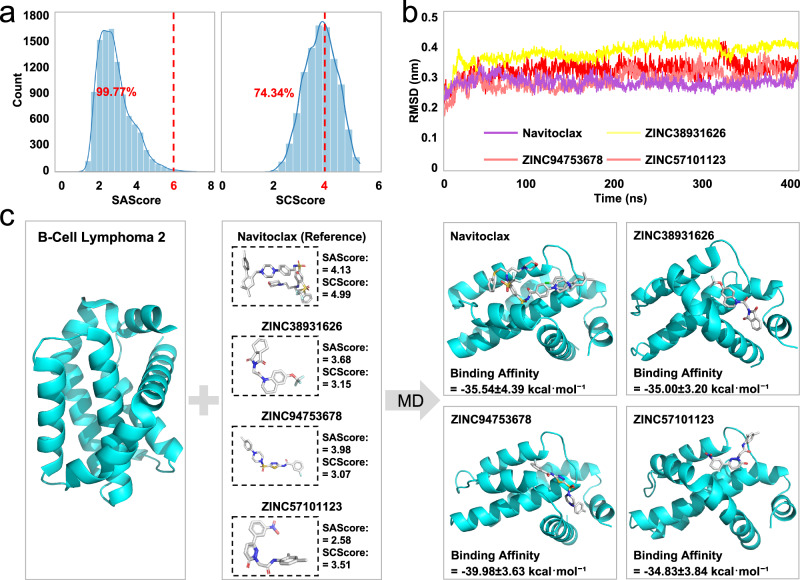
Virtual screening analysis and molecular dynamics simulation results. **a** Distributions of synthesizability metrics measured by Synthetic Accessibility Score (SAScore) and Synthetic Complexity Score (SCScore). For the top 10,000 ZINC candidates, empirical cutoffs (SAScore ≤ 6.0; SCScore ≤ 4.0) yield pass rates of 99.77%, 74.34%, and 74.17% for SAScore, SCScore, and both, respectively. **b** Root Mean Square Deviation (RMSD) analysis of the reference ligand (Navitoclax), ZINC38931626, ZINC94753678 and ZINC57101123 complexes. RMSD was plotted over 400 ns for Navitoclax and candidates. In all systems, ligand RMSD reaches a plateau after 100 ns and remains bounded within 0.20–0.35 nm, with no evidence of abrupt drift. **c** Representative Molecular Dynamics (MD) simulation results and corresponding binding affinities. MD simulations were performed with GROMACS^82^, and the resulting trajectories were used to estimate binding affinities (kcal·mol⁻¹) for the candidates. The candidates exhibit affinities comparable to the reference ligand, consistent with stable binding in the BCL-2 pocket.

BCL-2 is a clinically important anti-apoptotic protein and a well-established oncology target^57,58^. We assessed the top 10,000 candidates for BCL-2 as the receptor using two synthesizability metrics, SAScore (Synthetic Accessibility Score)^59^ and SCScore (Synthetic Complexity Score)^60^. SAScore ranges from 1 to 10 (higher values indicate harder synthesis), while SCScore ranges from 1 to 5 (higher values indicate greater synthetic complexity). Following prior benchmarks^61,62^, we applied cutoffs of SAScore ≤ 6.0 and SCScore ≤ 4, yielding pass rates of 99.77% (9977/10,000), 74.34% (7434/10,000), and 74.17% (7417/10,000) for SAScore, SCScore, and both thresholds, respectively. The corresponding distributions (Fig. 7a) indicate that the screened candidates are not biased toward exceptionally hard-to-synthesize compounds. Additionally, to further evaluate whether the same screened candidates used for docking (ZINC38931626, ZINC94753678, and ZINC57101123) can form stable complexes with BCL-2 beyond a static docking pose, we performed molecular dynamics (MD) simulations under explicit-solvent conditions to assess binding stability under thermal fluctuations^63–65^. Each complex was simulated for 400 ns using the TIP3P water model and the AMBER14SB force field. Ligand RMSD was monitored after alignment to the protein backbone to quantify conformational stability within the binding pocket (Fig. 7b). The candidates showed bounded RMSD trajectories without abrupt drift indicative of dissociation, and their RMSD profiles were comparable to that of the reference ligand over the simulation window. Representative bound conformations are shown in Fig. 7c, and binding strengths were further estimated using trajectory-based binding free-energy calculations with gmx_mmpbsa (reported as binding affinity in kcal·mol⁻¹). The candidates yielded favorable estimates consistent with stable binding to BCL-2.

## Discussion

The acquisition of high-quality experimental protein-ligand structures remains costly and environmentally sensitive, and a large fraction of available complexes are of modest resolution. Recent advances in structure prediction, most notably AlphaFold, have expanded access to in silico models but also increased heterogeneity in coordinate quality. Against this backdrop, we presented E-CloudBind, a structure-based learning framework that jointly models non-covalent fields and covalent topology. By replacing hard Euclidean cutoffs with electron-density point clouds, van-der-Waals-guided Gaussian clouds for protein pockets and semi-empirical densities for ligands, E-CloudBind embeds chemically meaningful neighborhood information and reduces sensitivity to coordinate noise.

Compared with methods that infer contacts using a single distance threshold, the density-adaptive representation yields more stable interaction graphs under perturbations and low-resolution inputs, improving generalization on out-of-distribution and low-quality structures. Empirically, E-CloudBind matches or exceeds strong sequence-, graph-, and structure-based baselines on PDBbind while showing minimal sensitivity to crystallographic resolution and to the source of 3D coordinates. Notably, performance on experimental versus AlphaFold structures differs by only ~0.012 MAE, and the error-resolution trend remains comparatively flat relative to baselines, indicating robustness to structural uncertainty.

Beyond accuracy, the model provides interpretable attention maps that align with known hydrogen-bond donors/acceptors and π-stacking residues in case studies, and it supports prospective virtual screening at scale. In our screens, many candidates achieved more favorable docking scores than co-crystallized ligands, highlighting chemotypes suitable for follow-up. While docking validation is inherently approximate, the agreement between attention hotspots and docking-derived interaction sites suggests that the learned density features capture salient non-covalent patterns relevant to medicinal chemistry.

This work underscores that learning on density, not distances, can make structure-based affinity prediction both chemically faithful and noise-tolerant. Limitations include the lack of explicit polarization and solvent effects in the current representation, as well as reliance on predefined pockets. Future directions include solvent-aware or polarization-augmented fields, end-to-end pocket discovery, and broader benchmarking across diverse target classes (e.g., kinases, GPCRs) to further assess generality. Extending the paradigm to pose scoring, selectivity, and target-guided molecular generation may enable transferable pre-trained DPI models on heterogeneous structural inputs.

## Methods

This section consists of three main subsections. First, Structural Preprocessing for Multi-Bond Interactions presents our method for transforming molecular and protein structures into electron-cloud-based representations, integrating covalent and non-covalent interaction information. Next, E-CloudBind presents the proposed model architecture. Finally, high-throughput screening and computational simulation configuration outlines the experimental setup for virtual screening across multiple biological targets.

### Structural preprocessing for multi-bond interactions

Traditional representations of molecular structures often rely on discrete 3D ball-and-stick models, which serve as classical approximations of molecular mechanics^35^. However, from the standpoint of theoretical physics, all intermolecular forces, such as hydrogen bonding, van der Waals interactions, and π-π stacking, are fundamentally governed by electromagnetic interactions and can be unified as manifestations of dipole-dipole coupling^66,67^. To better capture these underlying physical principles, we replace the rigid ball-and-stick framework with a more physically grounded electron-cloud representation^34^. This shift allows our model to directly learn from the continuous spatial distribution of electrons, thereby bypassing hand-crafted approximations and enhancing generalization. In practice, protein structures are obtained from diverse and often imperfect sources, including experimental resolutions, silicon-based predictions (e.g., AlphaFold), and varying instrumentation. Such heterogeneity introduces significant structural uncertainty. Current approaches to non-covalent interaction detection typically rely on a fixed Euclidean threshold (e.g., 5 Å), which is highly sensitive to structural deviations. This dependency can lead to unreliable assessments of interaction patterns, thereby limiting the robustness of many existing structure-based DPI models (Fig. 1a, c). In contrast, prior work^32,33,67^ has demonstrated that using electron-cloud structure as a prior can provide binding information that atom-level molecular graphs cannot fully capture, enabling a more direct characterization of non-covalent interactions. However, such methods often compress and replace the original 3D electron cloud via pretrained hierarchical codebooks or electron-density topological descriptors based on critical points (BCP/NCP). It inevitably degrades the shape and intensity distribution of the electron density in three-dimensional space. Moreover, because the representation is built on tokens or critical points, it often lacks explicit modeling of atom nodes and their covalent bond topology within the molecular graph. Therefore, motivated by the above considerations, we adopt the electron-cloud structure as the source of non-covalent interaction information, while simultaneously utilizing the intrinsic molecular graph, containing explicit chemical bonds, as the source of covalent interaction information.

### Electron-cloud structure for molecular non-covalent interactions

To evaluate the electron density distributions of molecular systems, electron clouds, representing the probability density of electrons, were computed as solutions to the time-independent Schrödinger equation^68^ under an effective potential framework:

1 $$\left[-\frac{1}{2}{\nabla }^{2}+{v}_{{\rm{eff}}}({{\rm{r}}})\right]{\phi }_{i}({{\rm{r}}})=\int _{i}{\phi }_{i}({{\rm{r}}})$$

Here, ${{\rm{r}}}$ denotes the position vector, ${\phi }_{i}(\cdot )$ is the molecular orbital wavefunction of the *i*-th electron state, ${\epsilon }_{i}$ is the corresponding eigenvalue, ${\nabla }^{2}$ is the Laplacian representing kinetic energy, and ${v}_{{{\rm{eff}}}}(\cdot )$ is the effective potential. The total electron density $\rho (\cdot )$ was obtained by summing over all occupied orbitals:

2 $$\rho ({{\rm{r}}})={\sum }_{i}|{\phi }_{i}({{\rm{r}}}){|}^{2}$$

Due to the high cost of ab initio methods, we adopted the GFN2-xTB model, a tight-binding approach with empirical corrections that offers reliable electron densities at significantly reduced computational expense compared to DFT^69,70^. GFN2-xTB calculations were based on the Cartesian coordinates $({x}_{i},{y}_{i},{z}_{i})$ of all ${N}_{L}$ atoms, which define the nuclear potential centers and guide the electron distribution. Electron densities were computed on a 64³ grid with a 0.5 Å spacing, yielding over a million low-energy density points under default convergence settings.

### Electron-like structure for protein non-covalent interactions

Given a protein with ${N}_{P}$ atoms, its atomic coordinates are denoted as ${{\rm{P}}}\in {{\mathbb{R}}}^{{N}_{P}\times 3}$. Due to the typically large number of atoms present in protein binding pockets (e.g., $N\approx {10}^{4}$), direct quantum-mechanical calculations of their electron density using GFN2-xTB are computationally prohibitive, as the interaction domain is extensive and molecular boundaries are often incomplete. Therefore, we propose a distance-based non-covalent interaction sampling modeling strategy based on van der Waals radii, which enables us to preserve chemically relevant local environments while avoiding full-system electronic calculations. This method leverages the definition of non-covalent interaction range and strength of atoms: (1) Non-covalent interaction force magnitude significantly declines by ${r}^{6}$ beyond the van der Waals radius^71^; (2) The closer to the central atom, the stronger the non-covalent interaction force, with the sampling density of the Gaussian kernel being defined based on the distance from the atomic nucleus to simulate the effect of the electron cloud^72–74^. We define the non-covalent interaction potential ${V}_{{{\rm{nc}}}}(\cdot )$ between atoms as follows:

For interatomic distances $r$ exceeding the van der Waals threshold ${r}_{c}$, non-covalent interactions decay exponentially with decay coefficient $\lambda$:

3 $${V}_{{{\rm{nc}}}}(r)\propto {e}^{-\lambda r},\,{{\rm{for}}}\; r > {r}_{c}$$

For shorter distances ($r < {r}_{c}$), where repulsive forces dominate, we approximate the strong short-range repulsion using a Gaussian function:

4 $${V}_{nc}(r)\propto \exp \left(-\frac{{(r-{r}_{0})}^{2}}{2{\sigma }^{2}}\right),\, \ {{\rm{for}}}\,r < {r}_{c}$$

Based on these definitions, we construct a probabilistic sampling distribution over a subset of atoms to capture the dominant non-covalent environment. Let ${{\rm{r}}}$ denote a spatial position. This is modeled as a 3D multivariate Gaussian distribution with mean position ${{\rm{\mu }}}$ and covariance matrix $\Sigma$:

5 $$\rho ({{\rm{r}}})=\frac{1}{{(2{{\pi }})}^{3/2}|\Sigma {|}^{1/2}}\exp \left(-\frac{1}{2}{({{\rm{r}}}-{{\rm{\mu }}})}^{\cdot }{\Sigma }^{-1}({{\rm{r}}}-{{\rm{\mu }}})\right)$$

Here, the mean ${{\rm{\mu }}}$ represents the geometric center of the selected atom subset, while $\Sigma$ is modeled as an isotropic diagonal matrix ${\sigma }^{2}{{\rm{I}}}$. This Gaussian sampling approach allows us to define a spatial region around atoms with high non-covalent potential, facilitating efficient fragment selection for subsequent quantum mechanical calculations. The probabilistic spatial distributions of different atom types are illustrated in Supplementary Fig. 2.

### Atomic features and intra-molecular covalent edges

Each protein-ligand complex is represented as a heterogeneous molecular graph ${{\mathcal{G}}}=({{{\mathcal{V}}}}_{L}\cup {{{\mathcal{V}}}}_{P},{\mathcal E} )$, where ${{{\mathcal{V}}}}_{L}$ and ${{{\mathcal{V}}}}_{P}$ denote the sets of ligand and protein atoms. The edge set ${\mathcal E}$ consists of covalent bonds within each molecular, and is partitioned into two subsets: ${ {\mathcal E} }_{L}=\{({v}_{i},{v}_{j})|{v}_{i},{v}_{j}\in {{{\mathcal{V}}}}_{L}\}$, indicating covalent bonds between ligand atoms, and ${ {\mathcal E} }_{P}=\{({v}_{i},{v}_{j})|{v}_{i},{v}_{j}\in {{{\mathcal{V}}}}_{P}\}$, indicating covalent bonds between protein atoms. Therefore, the complete edge set is ${\mathcal E}={ {\mathcal E} }_{L}\cup { {\mathcal E} }_{P}$. The final heterogeneous graph is encoded as:

6 $${{{\mathcal{G}}}}_{c}=\left\{\begin{array}{c}({{{\mathcal{V}}}}_{L},{ {\mathcal E} }_{L},{{{\rm{h}}}}_{i},{{{\rm{x}}}}_{ij})\\ ({{{\mathcal{V}}}}_{P},{ {\mathcal E} }_{P},{{{\rm{h}}}}_{i},{{{\rm{x}}}}_{ij})\end{array}\right\}$$

where the node feature ${{{\rm{h}}}}_{i}$ encodes atom-level chemical descriptors such as atom type, valence, hybridization state, aromaticity, while the edge feature ${{{\rm{x}}}}_{ij}$ represents covalent bond information between atom pairs.

### 3D point-cloud encoder for non-covalent feature

Let ${{{\mathcal{P}}}}_{L}={\{{{{\rm{p}}}}_{n}^{(L)}\in {{\mathbb{R}}}^{3}\}}_{n=1}^{{N}_{L}}$ and ${{{\mathcal{P}}}}_{P}={\{{{{\rm{p}}}}_{m}^{(P)}\in {{\mathbb{R}}}^{3}\}}_{m=1}^{{N}_{P}}$ denote the point clouds corresponding to the ligand and protein, respectively, where each point encodes a spatial location sampled from the electron density field. For the electron clouds ${{{\mathcal{P}}}}_{L}$ and ${{{\mathcal{P}}}}_{P}$, we define the number of clusters to match the corresponding number of atoms, ${N}_{L}$ and ${N}_{P}$, respectively.

The K-means clustering is applied separately to the electron-density point set of each molecule, producing one cluster per atom. Consistent with the QTAIM^75^ perspective that electron density features attractors associated with nuclei, clustering high-density regions yields centroids that tend to lie close to the corresponding atomic centers (i.e., near the true atomic positions). For the ligand, we compute the Euclidean distance between each centroid and all atomic coordinates, and assign each cluster to the atom with the minimum distance, establishing a one-to-one indexed correspondence between clusters and atoms. We then construct fixed-size local point sets by retaining the nearest points to each cluster center, so that every cluster yields a neighborhood of consistent shape. This allows us to unfold the atom dimension into the batch dimension for efficient batched training. Similarly, for the protein, since our proposed van der Waals Gaussian sampler uses each atom position as a proxy for the local electron distribution, it likewise supports an indexed cluster-atom alignment and the same fixed-size batching construction. The resulting ligand and protein vectors serve as the input to graph-based convolutional layers equipped with learnable deformable kernels ${{\mathcal{K}}}=\{{{{\rm{k}}}}_{C},{{{\rm{k}}}}_{1},\ldots,{{{\rm{k}}}}_{S}\}$, following 3D-GCN^76^. Within each cluster, the local geometric structure is captured by computing directional similarities between the electron clouds and their respective cluster centers. Specifically, ${{{\rm{k}}}}_{C}=0$ denotes the center of the learnable convolution kernel, and $\{{{{\rm{k}}}}_{1},\ldots,{{{\rm{k}}}}_{S}\}$ represent a set of learnable directional supports that enable the model to adaptively attend to local geometric patterns. Each support direction ${{{\rm{k}}}}_{s}$ is associated with a learnable filter weight ${{\rm{w}}}({{{\rm{k}}}}_{s})$, and the matching between the kernel and the local receptive field is performed based on directional and feature similarity. For example, considering an electron-cloud point ${{{\rm{p}}}}_{m}$ from the protein, within the neighborhood ${{{\mathcal{N}}}}_{m}$ of its corresponding cluster center ${{{\rm{p}}}}_{{\rm{clu}}}$, the directional similarity is computed as:

7 $${\mbox{sim}}({{{\rm{p}}}}_{m},{{{\rm{k}}}}_{s})=\frac{\langle {{\rm{f}}}({{{\rm{p}}}}_{m}),{{\rm{w}}}({{{\rm{k}}}}_{s})\rangle \cdot \langle {{{\rm{d}}}}_{m,{\mbox{clu}}},{{{\rm{k}}}}_{s}\rangle }{|{{{\rm{d}}}}_{m,{\mbox{clu}}}|\cdot |{{{\rm{k}}}}_{s}|},\,{{{\rm{d}}}}_{m,{\mbox{clu}}}={{{\rm{p}}}}_{m}-{{{\rm{p}}}}_{{\mbox{clu}}}$$

Here, ${{{\rm{d}}}}_{m,\,{\mbox{clu}}}$ denotes the relative spatial vector from the electron-cloud point to its cluster center, and ${{\rm{f}}}({{{\rm{p}}}}_{m})$ represents the feature vector of point ${{{\rm{p}}}}_{m}$. The similarity function ${\mbox{sim}}(\cdot )$ integrates both feature alignment and geometric directionality with respect to the learnable support direction ${{{\rm{k}}}}_{s}$. The convolution at a reference cluster center ${{{\rm{p}}}}_{{\mbox{clu}}}$ is computed as:

8 $${{\mbox{Conv}}}({{\mathcal{R}}}_{n},{{\mathcal{K}}})=\langle {{\rm{f}}}({{{\rm{p}}}}_{{\rm{clu}}}),{{\rm{w}}}({{{\rm{k}}}}_{C})\rangle+{\sum }_{s=1}^{S}{\max }_{{{{\rm{p}}}}_{m}\in {{{\mathcal{N}}}}_{m}}\{{{\rm{sim}}}({{{\rm{p}}}}_{m},{{{\rm{k}}}}_{s})\}$$

Here, the first term captures the feature response at the cluster center, while the second term aggregates the strongest directional similarities from the local electron clouds within the receptive field ${{{\mathcal{N}}}}_{m}$ for each learnable support direction ${{{\rm{k}}}}_{s}$.

After the convolutional operation, a pooling operation is applied to improve computational efficiency. Given the electron-cloud point sets ${{{\mathcal{P}}}}_{L}$ and ${{{\mathcal{P}}}}_{P}$, with their corresponding feature maps ${{{\rm{F}}}}_{L}={\{{{{\rm{f}}}}_{n}^{(L)}\}}_{n=1}^{{N}_{L}}$ and ${{{\rm{F}}}}_{P}={\{{{{\rm{f}}}}_{m}^{(P)}\}}_{m=1}^{{N}_{P}}$, we first perform per-channel max-pooling within each precomputed local neighborhood ${{{\mathcal{N}}}}_{i}$, where each point feature is smoothed as:

9 $${{{{\rm{f}}}}_{i}}={\max }_{j\in {{{\mathcal{N}}}}_{i}}\,{{{\rm{f}}}}_{j}$$

To reduce complexity, we then randomly sample subsets ${{{\mathcal{S}}}}_{L}\subset \{1,\ldots,{N}_{L}\}$ and ${{{\mathcal{S}}}}_{P}\subset \{1,\ldots,{N}_{P}\}$, each of size ${N}_{L}/{r}_{{{\rm{pool}}}}$ and ${N}_{P}/{r}_{{{\rm{pool}}}}$, respectively, where ${r}_{{{\rm{pool}}}}$ is the pooling ratio. The downsampled point clouds and corresponding features are:

10 $$\begin{array}{l}{{{\mathcal{P}}}}_{L}^{{\mbox{out}}}=\{{{{\rm{p}}}}_{n}^{(L)}|n\in {{{\mathcal{S}}}}_{L}\},{{{\rm{F}}}}_{L}^{{\mbox{out}}}=\{{\tilde{{{\rm{f}}}}}_{n}^{(L)}|n\in {{{\mathcal{S}}}}_{L}\}\\ {{{\mathcal{P}}}}_{P}^{{\mbox{out}}}=\{{{{\rm{p}}}}_{m}^{(P)}|m\in {{{\mathcal{S}}}}_{P}\},{{{\rm{F}}}}_{P}^{{\mbox{out}}}=\{{\tilde{{{\rm{f}}}}}_{m}^{(P)}|m\in {{{\mathcal{S}}}}_{P}\}\end{array}$$

Finally, after several layers of convolution and pooling, global max-pooling is applied over the downsampled ligand and protein features to obtain non-covalent representations:

11 $${{{\rm{f}}}}_{L}^{{\mbox{nc}}}={\max }_{n}\,{{\rm{f}}}({{{\rm{p}}}}_{n}^{(L)}),\,{{{\rm{f}}}}_{P}^{{\mbox{nc}}}={\max }_{m}\,{{\rm{f}}}({{{\rm{p}}}}_{m}^{(P)})$$

Here, ${{{\rm{f}}}}_{L}^{{\mbox{nc}}},{{{\rm{f}}}}_{P}^{{\mbox{nc}}}\in {{\mathbb{R}}}^{{d}_{{\mbox{nc}}}}$ are the final non-covalent feature representations derived from the 3D electron-cloud structures of the ligand and protein, respectively.

### Heterogeneous graph encoder for covalent interaction feature

In this work, we represent each protein-ligand complex as a heterogeneous graph consisting of two types of nodes and two types of intra-molecular covalent edges. Formally, the heterogeneous graph is defined as ${{\mathcal{G}}}=({{\mathcal{V}}},{\mathcal E} )$, along with a node type mapping function $\tau :{{\mathcal{V}}}\to {{\mathcal{A}}}$ and an edge type mapping function $\phi : {\mathcal E} \to {\mathcal R}$, where ${{\mathcal{A}}}=\{l,p\}$ denotes the set of node types (ligand $l$, protein $p$), and ${\mathcal R}=\{(l,l),(p,p)\}$ denotes the set of allowed covalent edge types. Edges are constructed based on chemically bonded pairs within the respective molecular structures. Specifically, for any edge ${e}_{ij}=({v}_{i},{v}_{j})\in {\mathcal E}$, the edge type is determined by:

12 $$\phi (({v}_{i},{v}_{j}))=\left\{\begin{array}{ll}(l,l),& {{\rm{if}}}\;\tau ({v}_{i})=l,\tau ({v}_{j})=l,\; {{\rm{and}}}\;{v}_{i}\;{{\rm{and}}}\;{v}_{j}\;{{\rm{share}}} \,{{\rm{a}}}\, {{\rm{covalent}}}\;{{\rm{bond}}}\\ (p,p),& {{\rm{if}}}\;\tau ({v}_{i})=p,\tau ({v}_{j})=p,{{\rm{and}}}\;{v}_{i}\;{{\rm{and}}}\;{v}_{j}\;{{\rm{share}}}\,{{\rm{a}}}\,{{\rm{covalent}}}\;{{\rm{bond}}}\end{array}\right.$$

Each atom node ${v}_{i}$ is initialized with a feature vector ${{{\rm{x}}}}_{i}\in {{\mathbb{R}}}^{n}$, which encodes atom-specific attributes. Similarly, each covalent bond ${e}_{ij}=({v}_{i},{v}_{j})$ is associated with a feature vector ${{{\rm{x}}}}_{ij}\in {{\mathbb{R}}}^{n{\prime} }$.

To learn contextualized node representations, we employ a message-passing graph neural network that iteratively updates each node's feature using both node and edge attributes. At the *t*-th layer, the hidden state of a node ${v}_{i}\in {{\mathcal{V}}}$ is denoted as ${{{\rm{h}}}}_{i}^{(t)}\in {{\mathbb{R}}}^{m}$, and the edge connecting nodes ${v}_{i}$ and ${v}_{j}$ is represented by ${{{\rm{h}}}}_{ij}^{(t)}\in {{\mathbb{R}}}^{m{\prime} }$. The node representation is updated according to the following message aggregation function:

13 $${{{\rm{h}}}}_{i}^{(t+1)}={g}_{\phi }^{(t)}\left({{{\rm{h}}}}_{i}^{(t)},{\sum }_{{v}_{j}\in {{\mathcal{N}}}({v}_{i})}{f}_{\theta }^{(t)}({{{\rm{h}}}}_{i}^{(t)},{{{\rm{h}}}}_{j}^{(t)},{{{\rm{h}}}}_{ij}^{(t)})\right)$$

where ${{\mathcal{N}}}({v}_{i})$ denotes the set of neighboring nodes of ${v}_{i}$, ${f}_{\theta }^{(t)}$ and ${g}_{\phi }^{(t)}$ are differentiable functions parameterized by learnable weights $\theta$ and $\phi$, respectively. The function ${f}_{\theta }^{(t)}$ computes pairwise messages using the local edge and node context, and ${g}_{\phi }^{(t)}$ aggregates them to update the target node state.

This message passing process is repeated for *T* iterations, resulting in final node embeddings ${{{\rm{h}}}}_{i}^{(T)}$ that capture rich chemical and topological context from local covalent structures. Since our heterogeneous graph contains two disjoint subgraphs (one for ligand atoms and one for protein atoms), the node representations are independently aggregated within each molecular component. Specifically, we apply a readout function (e.g., global average or max pooling) over each subgraph to obtain molecular-level embeddings:

14 $${{{\rm{z}}}}_{L}^{{\mbox{c}}}={{\mbox{Readout}}}\left(\left\{{{{\rm{h}}}}_{i}^{(T)}|\tau ({v}_{i})=l\right\}\right),\,{{{\rm{z}}}}_{P}^{{\mbox{c}}}={{\mbox{Readout}}}\left(\left\{{{{\rm{h}}}}_{i}^{(T)}|\tau ({v}_{i})=p\right\}\right)$$

The resulting embeddings ${{{\rm{z}}}}_{L}^{{\mbox{c}}}\in {{\mathbb{R}}}^{{d}_{c}}$ and ${{{\rm{z}}}}_{P}^{{\mbox{c}}}\in {{\mathbb{R}}}^{{d}_{{\mbox{c}}}}$ serve as the covalent feature representations of the ligand and protein.

### Multi-bond feature fusion for binding affinity prediction

Let the covalent interaction features be defined as ${{{\rm{f}}}}^{{\mbox{cov}}}=[{{{\rm{z}}}}_{L}^{{\mbox{c}}}||{{{\rm{z}}}}_{P}^{{\mbox{c}}}]\in {{\mathbb{R}}}^{2{d}_{{\mbox{c}}}}$, where ${{{\rm{z}}}}_{L}^{{\mbox{c}}}$ and ${{{\rm{z}}}}_{P}^{{\mbox{c}}}$ are the ligand and protein covalent representations obtained via message passing on the molecular graph. The corresponding non-covalent interaction features are defined as ${{{\rm{f}}}}^{{\rm{ncov}}}=[{{{\rm{f}}}}_{L}^{{\mbox{nc}}}||{{{\rm{f}}}}_{P}^{{\mbox{nc}}}]\in {{\mathbb{R}}}^{2{d}_{{\mbox{nc}}}}$, where ${{{\rm{f}}}}_{L}^{{\mbox{nc}}}$ and ${{{\rm{f}}}}_{P}^{{\mbox{nc}}}$ are derived from point-cloud convolution over 3D electron density fields.

To integrate complementary interaction features, we project the covalent representation into a shared space and concatenate it with the non-covalent features to form a unified multi-bond vector for binding affinity prediction. The final fused representation is expressed as:

15 $${{{\rm{f}}}}^{{\mbox{fused}}}=\left[{{\mbox{Dropout}}}\left({{{\rm{W}}}}_{q}\cdot \left[\begin{array}{c}{{{\rm{z}}}}_{L}^{c}\\ {{{\rm{z}}}}_{P}^{c}\end{array}\right]+{{{\rm{b}}}}_{q}\right)\,||\,\left[\begin{array}{c}{{{\rm{f}}}}_{L}^{{\mbox{nc}}}\\ {{{\rm{f}}}}_{P}^{{\mbox{nc}}}\end{array}\right]\right]\in {{\mathbb{R}}}^{1 \times (d+d{\prime} )}$$

Here, ${{{\rm{W}}}}_{q}$ and ${{{\rm{b}}}}_{q}$ are learnable parameters of the linear projection layer, and $d$,$d^{\prime}$ are the dimensions of the covalent and non-covalent subspaces, respectively.

Subsequently, the fused multi-bond interaction features are integrated into the protein-ligand graph ${{{\mathcal{G}}}}_{{{\rm{complex}}}}=({{\mathcal{V}}},{\mathcal E},\tau,\phi )$, where ${{\mathcal{V}}}$ denotes the set of atom nodes, ${\mathcal E}$ denotes the set of intra- and inter-molecular edges, $\tau$ and $\phi$ represent node-type and edge-type mappings, respectively. A heterogeneous graph neural network is employed to propagate messages across the graph by explicitly modeling relation-specific interactions.

To reduce information loss associated with bottlenecked graph encoders, we adopt a pairwise interaction modeling strategy inspired by EHIGN^30^, which explicitly computes atom-atom affinities between ligand atoms and protein atoms. For each ligand-protein atom pair $\phi ({e}_{ij})=(l,p)$, the interaction score is calculated using a learnable bilinear projection:

16 $${a}_{ij}^{(l,p)}={{{\rm{W}}}}_{{\rm{proj}}}(({{{\rm{W}}}}_{i}{{{\rm{h}}}}_{i}^{(T)})\circ ({{{\rm{W}}}}_{j}{{{\rm{h}}}}_{j}^{(T)})\circ ({{{\rm{W}}}}_{ij}{{{\rm{h}}}}_{ij}^{(T)}))$$

where ${{{\rm{W}}}}_{i}$,${{{\rm{W}}}}_{j}$,${{{\rm{W}}}}_{ij}\in {{\mathbb{R}}}^{m\times m}$ and ${{{\rm{W}}}}_{proj}\in {{\mathbb{R}}}^{1\times m}$ are learnable weight matrices, and $\circ$ denotes element-wise product. The overall protein-ligand binding affinity $\hat{y}$ is then computed by aggregating pairwise interactions in both directions:

17 $${\hat{y}}^{(l,p)}={\sum }_{\phi ({e}_{ij})=(l,p)}{a}_{ij}^{(l,p)},\,{\hat{y}}^{(p,l)}={\sum }_{\phi ({e}_{ij})=(p,l)}{a}_{ij}^{(p,l)},\,\hat{y}=\frac{1}{2}({\hat{y}}^{(l,p)}+{\hat{y}}^{(p,l)})$$

Meanwhile, to address structural bias introduced by varying numbers of atom pairs, the attention-based correction module is incorporated. Attention coefficients are assigned to each atom-atom pair based on fused embeddings:

18 $${c}_{ij}^{(l,p)}={{{\rm{W}}}}_{proj}(({{{\rm{W}}}}_{i}{{{\rm{h}}}}_{i}^{(T)})+({{{\rm{W}}}}_{j}{{{\rm{h}}}}_{j}^{(T)})+({{{\rm{W}}}}_{ij}{{{\rm{h}}}}_{ij}^{(T)})),\,{\alpha }_{ij}^{(l,p)}=\frac{\exp ({c}_{ij})}{{\sum }_{\phi ({e}_{uv})=(l,p)}\exp ({c}_{uv})}$$

These attention scores are used to compute a weighted aggregated representation, which is passed through an MLP ${f}_{\!\!\theta }$ to estimate a bias term:

19 $${\hat{b}}^{(l,p)}={f}_{\theta }\left(\mathop{\sum }_{\phi ({e}_{ij})=(l,p)}{\alpha }_{ij}\times ({{{\rm{W}}}}_{i}{{{\rm{h}}}}_{i}^{(T)}\circ {{{\rm{W}}}}_{j}{{{\rm{h}}}}_{j}^{(T)}\circ {{{\rm{W}}}}_{ij}{{{\rm{h}}}}_{ij}^{(T)})\right)$$

Finally, the corrected affinity score is computed by subtracting the averaged bias terms across both directions:

20 $${\hat{y}}_{c}=\hat{y}-\frac{1}{2}({\hat{b}}^{(l,p)}+{\hat{b}}^{(p,l)})$$

Additionally, to obtain the contribution of each ligand atom, the pair-wise attention weights $\alpha$ are aggregated to ligand nodes by summing the attention weights of ligand to pocket edges originating from ligand atom and pocket to ligand edges terminating at ligand atom, and then adding the two directional sums to yield a per-atom importance vector in ${{\mathbb{R}}}^{{N}_{L}}$. Atoms with no incident cross-molecule edges are assigned a score of zero.

### Experimental setup

#### Datasets

In this study, three commonly used datasets were employed to support different experimental objectives in drug-protein interaction prediction and drug discovery. First, PDBbind^42,43^ was used as the primary structure-based affinity benchmark to evaluate the E-Cloudbind’s ability to learn binding interactions from experimentally resolved protein-ligand complexes. Specifically, different PDBbind subsets were used for model training and systematic evaluation, including resolution robustness analysis, structural robustness assessment across experimental and AlphaFold2 structures^23^, ablation studies, and cold-start scenario analysis. Second, DAVIS^47^ was used as a kinase-inhibitor binding affinity dataset to evaluate E-Cloudbind’s performance in complexity-based robustness evaluation and cross-source structural generalization analysis. Finally, ZINC^55^ was used for large-scale virtual screening and candidate compound prioritization, providing a chemically diverse compound library for downstream drug discovery applications. Detailed descriptions of these datasets are provided in the Dataset section of the Supplementary Information.

#### Baselines

We first chose DMFF^46^, PSICHIC^20^^,^ and SIGN^17^ as representative baseline models for different input modalities. These models reflect typical paradigms in current DPI prediction, including sequence-based, graph-based, and geometry-based modeling, and were used in all subsequent experiments. In addition to these baseline models, we also introduce representative advanced structural modeling methods, including EHIGN^30^, FlowDock^45^, and Boltz-2^44^, for supplementary comparisons. Detailed descriptions of these datasets are provided in the Benchmarking Against State-of-the-Art Methods section of the Supplementary Information.

#### Training details

For software implementation, E-CloudBind was trained and evaluated using PyTorch (1.12.1) with CUDA (11.3). The main Python packages included DGL-CUDA (0.9.1.post1), NumPy (1.23.5), Pandas (2.0.3), SciPy (1.9.0), scikit-learn (1.2.2), RDKit (2024.3.3) and Biopython (1.83). Additional packages used for data processing, visualization, and structure preprocessing included Matplotlib (3.7.5), Seaborn (0.13.2), Open Babel (3.1.1), PyMOL (2.4.1), OpenPyXL (3.1.5), and Requests (2.32.3). During the affinity-fitting phase, E-Cloudbind was optimized using the mean-squared-error loss function. Details of the hardware configuration and model architecture are provided in the Training Details and Model Hyperparameters section of the Supplementary Information.

### High-throughput screening and docking configuration

#### High-throughput screening configuration

We utilized the core set from ZINC-250K, comprising 100,000 commercially available small-molecule compounds. The electron density of each molecule was first estimated using RDKit in combination with the semi-empirical quantum chemical method GFN2-xTB. Based on the spatial distribution of electronic points, a K-means clustering algorithm was applied to cluster electron-cloud points into localized groups, enabling the construction of representations of non-covalent interaction potential. For the target proteins, we selected three structurally diverse and pharmacologically significant receptors: 4OON, 7RFS, and 4MAN, each associated with distinct diseases and molecular binding mechanisms. These targets were chosen to assess the model's robustness and generalizability across varying structural complexities and binding modalities.

- 4OON encodes the Penicillin-Binding Protein 1 A (PBP1A) from Escherichia coli, a vital component of bacterial cell wall synthesis^77^. As a key target of $\beta$-lactam antibiotics, PBPs play a crucial role in antimicrobial drug discovery. Mutations or inhibition of PBP1A disrupts peptidoglycan biosynthesis, making it a cornerstone target in combating antibiotic-resistant infections.
- 7RFS corresponds to the SARS-CoV-2 Main Protease, which is critical for viral polyprotein processing and replication^78^. As an essential viral enzyme, Mpro has emerged as a frontline target for therapeutic intervention in the treatment of COVID-19. Its structure-guided inhibition represents one of the most promising strategies in pandemic drug development.
- 4MAN represents the B-Cell Lymphoma 2 (BCL-2) protein in complex with the selective inhibitor ABT-199 (venetoclax)^79^. BCL-2 is an anti-apoptotic protein that plays a pivotal role in cell survival by inhibiting the mitochondrial apoptotic pathway. Overexpression of BCL-2 is frequently observed in various cancers, particularly hematological malignancies, and contributes to tumor cell resistance to apoptosis. The structural elucidation has guided the development of venetoclax, a highly selective BCL-2 inhibitor with potent antitumor activity and reduced platelet toxicity.

We estimated binding affinities for each of the three proteins and all molecules in the ZINC subset using our trained framework. The top 5% of protein-ligand pairs, ranked by predicted binding affinity, were then selected for molecular docking simulations. The resulting complexes were visualized with a focus on non-covalent interactions.

#### Docking configuration

Molecular docking was carried out using AutoDock Vina (1.2.7)^80^ to evaluate the binding affinities of the generated compounds to their respective protein targets. Semiflexible docking was employed, allowing full conformational flexibility of the ligands while maintaining the receptor structures rigid. For each target, the docking grid was centered on the geometric centroid of the co-crystallized ligand, calculated as the average position of all heavy atoms. The grid box dimensions were determined by expanding the maximal extent of the ligand by 5 Å in each Cartesian direction, ensuring comprehensive coverage of the binding pocket. Receptor structures were prepared by removing crystallographic water molecules (unless stated otherwise), adding polar hydrogen atoms, and assigning Gasteiger partial charges using AutoDockTools. Ligand structures were geometry-optimized and converted to the appropriate format using Open Babel^81^. Docking results were ranked by Vina’s scoring function (reported in kcal mol^-1^), with the top-scoring binding poses subjected to further visual inspection to assess their spatial alignment with known interaction motifs.

#### Molecular dynamics simulations

The single peptide-small molecule systems were placed at the center of a cubic box with dimensions of 6 × 6 × 6 nm. In contrast, the multicomponent systems were placed in a larger 15 × 15 × 15 nm cubic box, with ten peptide molecules and ten small molecules randomly inserted. The relative positions of the peptide molecules within each system were kept consistent. Additionally, a separate simulation system was constructed for the ten peptide molecules, where their relative positions matched those in the multicomponent systems.

All molecular dynamics (MD) simulations were performed using the GROMACS 2022.5 package with periodic boundary conditions^82^. The peptides were modeled with the Amber14SB all-atom force field, while the small molecules were modeled with the GAFF force field, combined with the TIP3P water model^83–85^. Long-range electrostatics were computed using the Particle Mesh Ewald (PME) method, with a real-space cutoff of 1.0 nm and fourth-order spline interpolation. A 1.0 nm cutoff was applied for van der Waals interactions. Hydrogen bonds were constrained using the LINCS algorithm^86^. The system temperature was maintained at 300 K using the V-rescale method^87^, while the pressure was kept at 1 bar using the C-rescale method^88^. Each system underwent energy minimization via the steepest descent method and was equilibrated for 100 ps in the NVT ensemble at 300 K. Production runs were conducted for 150 ns in the NPT ensemble. A time step of 2 fs was used, with trajectory snapshots saved every 20 ps, resulting in a total of 7,500 frames for subsequent analysis. Built-in GROMACS tools were employed to analyze the system’s RMSD, time-dependent SASA variations, and hydrogen-bonding interactions between the peptides and small molecules. The gmx_MMPBSA tool was used to calculate the binding energies of the complexes^89^. Visualization of the simulations was performed using VMD^90^.

## Supplementary information

Supplementary Information

Description of Additional Supplementary Files

Supplementary Data 1

Transparent Peer Review file

## Source data

Source Data

## Data Availability

All datasets used in our study are open to public and can be accessed through cited references. The PDBbind dataset used in this study is available at https://www.pdbbind-plus.org.cn/, the ZINC dataset is available at https://zinc.docking.org/, and the DAVIS dataset is available at https://github.com/dingyan20/Davis-Dataset-for-DTA-Prediction. The source code of E-CloudBind is available at https://github.com/Liuyujian0408/DPI. The processed data repository can be accessed 10.6084/m9.figshare.30343492 on figshare^91^, which includes the predefined training/validation set and test set. Source data are provided with this paper.

## Code availability

The source code of this work can be accessed via the GitHub repository at https://github.com/Liuyujian0408/DPI or the Zenodo repository at 10.5281/zenodo.19851869^92^.

## References

[1] Dara, S. et al. Machine learning in drug discovery: a review. *Artif. Intell. Rev.* **55**, 1947–1999 (2022). 34393317 10.1007/s10462-021-10058-4 PMC8356896

[2] Luo, Y. et al. A network integration approach for drug-target interaction prediction and computational drug repositioning from heterogeneous information. *Nat. Commun.* **8**, 573 (2017). 28924171 10.1038/s41467-017-00680-8 PMC5603535

[3] Yamanishi, Y. et al. Prediction of drug-target interaction networks from the integration of chemical and genomic spaces. *Bioinformatics* **24**, i232–i240 (2008). 18586719 10.1093/bioinformatics/btn162 PMC2718640

[4] Öztürk, H., Özgür, A. & Ozkirimli, E. DeepDTA: deep drug-target binding affinity prediction. *Bioinformatics* **34**, i821–i829 (2018). 30423097 10.1093/bioinformatics/bty593 PMC6129291

[5] Gilson, M. K. et al. BindingDB in 2015: a public database for medicinal chemistry, computational chemistry and systems pharmacology. *Nucleic Acids Res.* **44**, D1045–D1053 (2016). 26481362 10.1093/nar/gkv1072 PMC4702793

[6] Kitchen, D. B. et al. Docking and scoring in virtual screening for drug discovery: methods and applications. *Nat. Rev. Drug Discov.* **3**, 935–949 (2004). 15520816 10.1038/nrd1549

[7] Zhu, H. et al. A pharmacophore-guided deep learning approach for bioactive molecular generation. *Nat. Commun.* **14**, 6234 (2023). 37803000 10.1038/s41467-023-41454-9 PMC10558534

[8] Goodnow, R. A. Jr Hit and lead identification: integrated technology-based approaches. *Drug Discov. Today.: Technol.* **3**, 367–375 (2006).

[9] Gao K. Y., et al. Interpretable drug target prediction using deep neural representation. In: *Proc. 27th International Joint Conference on Artificial Intelligence*. pp. 3371–3377 (IJCAI, 2018).

[10] Chen, L. et al. TransformerCPI: improving compound-protein interaction prediction by sequence-based deep learning with self-attention mechanism and label reversal experiments. *Bioinformatics* **36**, 4406–4414 (2020). 32428219 10.1093/bioinformatics/btaa524

[11] Jiang, M. et al. Drug-target affinity prediction using graph neural network and contact maps. *RSC Adv.* **10**, 20701–20712 (2020). 35517730 10.1039/d0ra02297g PMC9054320

[12] Li S., et al. Structure-aware interactive graph neural networks for the prediction of protein-ligand binding affinity. In: *Proc. 27th ACM SIGKDD Conference on Knowledge Discovery & Data Mining*. 975–985 (SIGKDD, 2021).

[13] Bai, P. et al. Interpretable bilinear attention network with domain adaptation improves drug-target prediction. *Nat. Mach. Intell.* **5**, 126–136 (2023).

[14] Huang, K. et al. MolTrans: molecular interaction transformer for drug-target interaction prediction. *Bioinformatics* **37**, 830–836 (2021). 33070179 10.1093/bioinformatics/btaa880 PMC8098026

[15] Nguyen, T. et al. GraphDTA: predicting drug-target binding affinity with graph neural networks. *Bioinformatics* **37**, 1140–1147 (2021). 33119053 10.1093/bioinformatics/btaa921

[16] Tsubaki, M., Tomii, K. & Sese, J. Compound-protein interaction prediction with end-to-end learning of neural networks for graphs and sequences. *Bioinformatics* **35**, 309–318 (2019). 29982330 10.1093/bioinformatics/bty535

[17] Jiang, D. et al. Interactiongraphnet: a novel and efficient deep graph representation learning framework for accurate protein–ligand interaction predictions. *J. Med. Chem.* **64**, 18209–18232 (2021). 34878785 10.1021/acs.jmedchem.1c01830

[18] Luo, Y., Liu, Y. & Peng, J. Calibrated geometric deep learning improves kinase-drug binding predictions. *Nat. Mach. Intell.* **5**, 1390–1401 (2023). 38962391 10.1038/s42256-023-00751-0 PMC11221792

[19] Sun C., et al. DCGCN: dual-channel graph convolutional network-based drug-target interaction prediction method with 3D molecular structure. *J. Chem. Inf. Model.* **65**, 7529–7539 (2025). 10.1021/acs.jcim.5c01012 40605230

[20] Koh, H. Y. et al. Physicochemical graph neural network for learning protein-ligand interaction fingerprints from sequence data. *Nat. Mach. Intell.* **6**, 673–687 (2024).

[21] Karimi, M. et al. DeepAffinity: interpretable deep learning of compound-protein affinity through unified recurrent and convolutional neural networks. *Bioinformatics* **35**, 3329–3338 (2019). 30768156 10.1093/bioinformatics/btz111 PMC6748780

[22] Jones, D. et al. Improved protein-ligand binding affinity prediction with structure-based deep fusion inference. *J. Chem. Inf. Model.* **61**, 1583–1592 (2021). 33754707 10.1021/acs.jcim.0c01306

[23] Jumper, J. et al. Highly accurate protein structure prediction with AlphaFold. *Nature* **596**, 583–589 (2021). 34265844 10.1038/s41586-021-03819-2 PMC8371605

[24] Abramson, J. et al. Accurate structure prediction of biomolecular interactions with AlphaFold 3. *Nature* **630**, 493–500 (2024). 38718835 10.1038/s41586-024-07487-w PMC11168924

[25] Wong, F. et al. Benchmarking AlphaFold-enabled molecular docking predictions for antibiotic discovery. *Mol. Syst. Biol.* **18**, e11081 (2022). 36065847 10.15252/msb.202211081 PMC9446081

[26] He, X. et al. AlphaFold2 versus experimental structures: evaluation on G protein-coupled receptors. *Acta Pharmacologica Sin.* **44**, 1–7 (2023). 10.1038/s41401-022-00938-y PMC9813356 35778488

[27] Tunyasuvunakool, K. et al. Highly accurate protein structure prediction for the human proteome. *Nature* **596**, 590–596 (2021). 34293799 10.1038/s41586-021-03828-1 PMC8387240

[28] Bagherian, M. et al. Machine learning approaches and databases for prediction of drug-target interaction: a survey paper. *Brief. Bioinforma.* **22**, 247–269 (2021). 10.1093/bib/bbz157 PMC7820849 31950972

[29] Kyro, G. W. et al. T-ALPHA: a hierarchical transformer-based deep neural network for protein-ligand binding affinity prediction with uncertainty-aware self-learning for protein-specific alignment. *J. Chem. Inf. Model.* **65**, 2395–2415 (2025). 39965912 10.1021/acs.jcim.4c02332

[30] Yang, Z. et al. Interaction-based inductive bias in graph neural networks: enhancing protein-ligand binding affinity predictions from 3D structures. *IEEE Trans. Pattern Anal. Mach. Intell.* **46**, 8191–8208 (2024). 38739515 10.1109/TPAMI.2024.3400515

[31] Volkov, M. et al. On the frustration to predict binding affinities from protein-ligand structures with deep neural networks. *J. Med. Chem.* **65**, 7946–7958 (2022). 35608179 10.1021/acs.jmedchem.2c00487

[32] Lin, H. et al. Tokenizing electron cloud in protein-ligand interaction learning. ArXiv Preprint ArXiv:2505.19014, 2025.

[33] Isert, C. et al. Exploring protein-ligand binding affinity prediction with electron density-based geometric deep learning. *RSC Adv.* **14**, 4492–4502 (2024). 38312732 10.1039/d3ra08650j PMC10835705

[34] Sebens, C. T. Electron charge density: A clue from quantum chemistry for quantum foundations. *Found. Phys.* **51**, 75 (2021).

[35] Hinze, S. R. et al. Beyond ball-and-stick: students' processing of novel STEM visualizations. *Learn. Instr.* **26**, 12–21 (2013).

[36] Skogh, M. et al. The electron density: a fidelity witness for quantum computation. *Chem. Sci.* **15**, 2257–2265 (2024). 38332826 10.1039/d3sc05269a PMC10848700

[37] Nakatsuji, H. Electron-cloud following and preceding and the shapes of molecules. *J. Am. Chem. Soc.* **96**, 30–37 (1974).

[38] Senior, A. W. et al. Improved protein structure prediction using potentials from deep learning. *Nature* **577**, 706–710 (2020). 31942072 10.1038/s41586-019-1923-7

[39] Ingraham, J. B. et al. Illuminating protein space with a programmable generative model. *Nature* **623**, 1070–1078 (2023). 37968394 10.1038/s41586-023-06728-8 PMC10686827

[40] Ahdritz, G. et al. OpenFold: retraining AlphaFold2 yields new insights into its learning mechanisms and capacity for generalization. *Nat. Methods* **21**, 1514–1524 (2024). 38744917 10.1038/s41592-024-02272-z PMC11645889

[41] Lin, Z. et al. Evolutionary-scale prediction of atomic-level protein structure with a language model. *Science* **379**, 1123–1130 (2023). 36927031 10.1126/science.ade2574

[42] Wang, R. et al. The PDBbind database: collection of binding affinities for protein-ligand complexes with known three-dimensional structures. *J. Med. Chem.* **47**, 2977–2980 (2004). 15163179 10.1021/jm030580l

[43] Liu, Z. et al. PDB-wide collection of binding data: current status of the PDBbind database. *Bioinformatics* **31**, 405–412 (2015). 25301850 10.1093/bioinformatics/btu626

[44] Passaro S., et al. Boltz-2: towards accurate and efficient binding affinity prediction. BioRxiv, 2025.

[45] Morehead, A. & Cheng, J. FlowDock: geometric flow matching for generative protein-ligand docking and affinity prediction. *Bioinformatics* **41**, i198–i206 (2025). 40662794 10.1093/bioinformatics/btaf187 PMC12261468

[46] He, H. et al. Dual modality feature-fused neural network integrating binding site information for drug target affinity prediction. *npj Digit. Med.* **8**, 67 (2025). 39875637 10.1038/s41746-025-01464-x PMC11775287

[47] Davis, M. I. et al. Comprehensive analysis of kinase inhibitor selectivity. *Nat. Biotechnol.* **29**, 1046–1051 (2011). 22037378 10.1038/nbt.1990

[48] Plaxco, K. W., Simons, K. T. & Baker, D. Contact order, transition state placement and the refolding rates of single domain proteins. *J. Mol. Biol.* **277**, 985–994 (1998). 9545386 10.1006/jmbi.1998.1645

[49] Özçelik, R. et al. Chemical language modeling with structured state space sequence models. *Nat. Commun.* **15**, 6176 (2024). 39039051 10.1038/s41467-024-50469-9 PMC11263548

[50] Durairaj J., et al. PLINDER: the protein-ligand interactions dataset and evaluation resource. *Workshop ML for Life and Material Science: From Theory to Industry Applications*. (ICML, 2024)

[51] Betzi, S. et al. Discovery of a potential allosteric ligand binding site in CDK2. *ACS Chem. Biol.* **6**, 492–501 (2011). 21291269 10.1021/cb100410m PMC3098941

[52] Wang, F. et al. Discovery of potent 2-Aryl-6, 7-dihydro-5 H-pyrrolo [1, 2-a] imidazoles as WDR5-WIN-site inhibitors using fragment-based methods and structure-based design. *J. Med. Chem.* **61**, 5623–5642 (2018). 29889518 10.1021/acs.jmedchem.8b00375 PMC6842305

[53] Deakyne J. S., et al. Structural and functional basis for an EBNA1 hexameric ring in Epstein-Barrs episome maintenance. *J. Virol.* **91**, 01046-17 (2017). 10.1128/JVI.01046-17 PMC5599765 28701406

[54] Conti, E. et al. X-ray and spectrophotometric studies of the binding of proflavin to the S1 specificity pocket of human α-thrombin. *FEBS Lett.* **425**, 229–233 (1998). 9559654 10.1016/s0014-5793(98)00235-x

[55] Sterling, T. & Irwin, J. J. ZINC 15-ligand discovery for everyone. *J. Chem. Inf. Model.* **55**, 2324–2337 (2015). 26479676 10.1021/acs.jcim.5b00559 PMC4658288

[56] Bento, A. P. et al. An open-source chemical structure curation pipeline using RDKit. *J. Cheminf.* **12**, 51 (2020). 10.1186/s13321-020-00456-1 PMC7458899 33431044

[57] Youle, R. J. & Strasser, A. The BCL-2 protein family: opposing activities that mediate cell death. *Nat. Rev. Mol. Cell Biol.* **9**, 47–59 (2008). 18097445 10.1038/nrm2308

[58] Birkinshaw, R. W. et al. Structures of BCL-2 in complex with venetoclax reveal the molecular basis of resistance mutations. *Nat. Commun.* **10**, 2385 (2019). 31160589 10.1038/s41467-019-10363-1 PMC6547681

[59] Ertl, P. & Schuffenhauer, A. Estimation of synthetic accessibility score of drug-like molecules based on molecular complexity and fragment contributions. *J. Cheminf.* **1**, 8 (2009). 10.1186/1758-2946-1-8 PMC3225829 20298526

[60] Coley, C. W. et al. SCScore: synthetic complexity learned from a reaction corpus. *J. Chem. Inf. Model.* **58**, 252–261 (2018). 29309147 10.1021/acs.jcim.7b00622

[61] Voršilák, M. et al. SYBA: Bayesian estimation of synthetic accessibility of organic compounds. *J. Cheminf.* **12**, 35 (2020). 10.1186/s13321-020-00439-2 PMC7238540 33431015

[62] Kim, J. et al. SHARP: generating synthesizable molecules via fragment-based hierarchical action-space reinforcement learning for Pareto optimization. *J. Chem. Inf. Model.* **65**, 11601–11619 (2025). 41134962 10.1021/acs.jcim.5c01699 PMC12606630

[63] Karplus, M. & McCammon, J. A. Molecular dynamics simulations of biomolecules. *Nat. Struct. Biol.* **9**, 646–652 (2002). 12198485 10.1038/nsb0902-646

[64] Lionta, E. et al. Structure-based virtual screening for drug discovery: principles, applications and recent advances. *Curr. Top. Med. Chem.* **14**, 1923–1938 (2014). 25262799 10.2174/1568026614666140929124445 PMC4443793

[65] Vasiliauskaité-Brooks, I. et al. Structure of a human intramembrane ceramidase explains enzymatic dysfunction found in leukodystrophy. *Nat. Commun.* **9**, 5437 (2018). 30575723 10.1038/s41467-018-07864-w PMC6303388

[66] Leckband, D. & Israelachvili, J. Intermolecular forces in biology. *Q. Rev. Biophys.* **34**, 105–267 (2001). 11771120 10.1017/s0033583501003687

[67] Zhang, O. et al. ECloudGen: leveraging electron clouds as a latent variable to scale up structure-based molecular design. *Nat. Comput. Sci*., **5**, 1017–1028 (2025). 10.1038/s43588-025-00886-7 41094041

[68] Schrödinger, E. An undulatory theory of the mechanics of atoms and molecules. *Phys. Rev.* **28**, 1049 (1926).

[69] Bannwarth, C. et al. Extended tight-binding quantum chemistry methods. *Wiley Interdiscip. Rev.: Comput. Mol. Sci.* **11**, e1493 (2021).

[70] Bannwarth, C., Ehlert, S. & Grimme, S. GFN2-xTB-an accurate and broadly parametrized self-consistent tight-binding quantum chemical method with multipole electrostatics and density-dependent dispersion contributions. *J. Chem. Theory Comput.* **15**, 1652–1671 (2019). 30741547 10.1021/acs.jctc.8b01176

[71] Leach A. R. *Molecular Modelling: Principles and Applications.* (Pearson Education, 2001).

[72] Bondi, A. van der Waals volumes and radii. *J. Phys. Chem.* **68**, 441–451 (1964).

[73] Klein, R. A. Modified van der Waals atomic radii for hydrogen bonding based on electron density topology. *Chem. Phys. Lett.* **425**, 128–133 (2006).

[74] Dauparas J., et al. Atomic context-conditioned protein sequence design using LigandMPNN. *Nat. Methods* **22**, 717–723 (2025). 10.1038/s41592-025-02626-1 PMC11978504 40155723

[75] Bader, R. F. W. Atoms in molecules. *Acc. Chem. Res.* **18**, 9–15 (1985).

[76] Lin Z. H., Huang S. Y., Wang Y. C. F. Convolution in the cloud: learning deformable kernels in 3d graph convolution networks for point cloud analysis. In: *Proc. IEEE/CVF Conference on Computer Vision and Pattern Recognition*. pp. 1800–1809 (CVPR, 2020).

[77] Starr, J. et al. Siderophore receptor-mediated uptake of lactivicin analogues in gram-negative bacteria. *J. Med. Chem.* **57**, 3845–3855 (2014). 24694215 10.1021/jm500219c

[78] Owen, D. R. et al. An oral SARS-CoV-2 Mpro inhibitor clinical candidate for the treatment of COVID-19. *Science* **374**, 1586–1593 (2021). 34726479 10.1126/science.abl4784

[79] Souers, A. J. et al. ABT-199, a potent and selective BCL-2 inhibitor, achieves antitumor activity while sparing platelets. *Nat. Med.* **19**, 202–208 (2013). 23291630 10.1038/nm.3048

[80] Trott, O. & Olson, A. J. AutoDock Vina: improving the speed and accuracy of docking with a new scoring function, efficient optimization, and multithreading. *J. Comput. Chem.* **31**, 455–461 (2010). 19499576 10.1002/jcc.21334 PMC3041641

[81] O'Boyle, N. M. et al. Open Babel: an open chemical toolbox. *J. Cheminf.* **3**, 33 (2011). 10.1186/1758-2946-3-33 PMC3198950 21982300

[82] Van Der Spoel, D. et al. GROMACS: fast, flexible, and free. *J. Comput. Chem.* **26**, 1701–1718 (2005). 16211538 10.1002/jcc.20291

[83] Maier, J. A. et al. ff14SB: improving the accuracy of protein side chain and backbone parameters from ff99SB. *J. Chem. Theory Comput.* **11**, 3696–3713 (2015). 26574453 10.1021/acs.jctc.5b00255 PMC4821407

[84] Wang, J., Wolf, R. M., Caldwell, J. W., Kollman, P. A. & Case, D. A. Development and testing of a general amber force field. *J. Comput. Chem.* **25**, 1157–1174 (2004). 15116359 10.1002/jcc.20035

[85] Jorgensen, W. L., Chandrasekhar, J., Madura, J. D., Impey, R. W. & Klein, M. L. Comparison of simple potential functions for simulating liquid water. *J. Chem. Phys.* **79**, 926–935 (1983).

[86] Hess, B., Bekker, H., Berendsen, H. J. C. & Fraaije, J. G. E. M. LINCS: A linear constraint solver for molecular simulations. *J. Comput. Chem.* **18**, 1463–1472 (1997).

[87] Berendsen, H. J. C., Postma, J. P. M., Van Gunsteren, W. F., DiNola, A. & Haak, J. R. Molecular dynamics with coupling to an external bath. *J. Chem. Phys.* **81**, 3684–3690 (1984).

[88] Bernetti, M. & Bussi, G. Pressure control using stochastic cell rescaling. *J. Chem. Phys.* **153**, 114107 (2020). 10.1063/5.0020514 32962386

[89] Valdés-Tresanco, M. S., Valdés-Tresanco, M. E., Valiente, P. A. & Moreno, E. gmx_MMPBSA: a new tool to perform end-state free energy calculations with GROMACS. *J. Chem. Theory Comput.* **17**, 6281–6291 (2021). 34586825 10.1021/acs.jctc.1c00645

[90] Humphrey, W., Dalke, A. & Schulten, K. VMD: visual molecular dynamics. *J. Mol. Graph.* **14**, 33–38 (1996). 8744570 10.1016/0263-7855(96)00018-5

[91] Liu, W. et al. An electron-density point-cloud framework for robust protein-ligand interaction prediction. *ECloudBind_data*10.6084/m9.figshare.30343492 (2026). 10.1038/s41467-026-74196-5 PMC13408660 42277055

[92] Liu, W. et al. An electron-density point-cloud framework for robust protein-ligand interaction prediction. *DPI: E-CloudBind*10.5281/zenodo.19851869 (2026). 10.1038/s41467-026-74196-5 PMC13408660 42277055

