# Supplementary material for: An electron-density point-cloud framework for robust protein-ligand interaction prediction
Source: Nat Commun. 2026 Jun 11;17:7424. doi: 10.1038/s41467-026-74196-5 (PMC13408660; doi:10.1038/s41467-026-74196-5)
Supplement: Supplementary file 1 — Supplementary Information [file 41467_2026_74196_MOESM1_ESM.pdf]

1 Training Details and Model Hyperparameters

Supplementary Table 1. Training Setting and Model Configuration.

	Hyper-parameters	Value
Training Setting	Learning rate	0.0001
	Weight decay	0.00001
	Batch size	48
	Epochs	500
	Early stop epoch	60
	Fold	10
	Drop out	0.1
Model Configuration	Node feature size	35
	Edge feature size	17
	Hidden feature size	256
	Layer number	1

E-CloudBind contains 1.79 × 10^6 learnable parameters. As shown in **Supplementary Table 1**, the model was trained on a system running Ubuntu 20.04.6 LTS, utilizing both NVIDIA GeForce RTX 3090 and RTX 4090 GPUs to ensure efficient computation. The training process was configured with a learning rate of 0.0001 and a batch size of 48. We trained the model for a maximum of 500 epochs, implementing an early stopping criterion of 60 epochs to prevent overfitting, which halts training if no improvement in validation loss is observed. A dropout rate of 0.1 was also applied for regularization. As shown in **Supplementary Table 2,** the architectural details of the model are provided, including its input and output dimensions and the purpose and functionality of each module.

Supplementary Table 2. Model Architecture.

<table>
<tr><th>Module Name</th><th>Input</th><th>Output</th><th>Process</th><th>Note</th></tr>
<tr><td rowspan="4">3D-GCN</td><td>$(B_1, N, 3)$</td><td>-</td><td>Point-cloud coordinates</td><td rowspan="4">Directional kernel num=32
Pooling rate=4
Nearest neighbor num=5
Support num=1
B_1 denotes the total number of protein B_1^{pocket} or ligand B_1^{ligand} atoms across all molecules in the current batch.
N represents the number of electron-cloud points associated with each atom following KNN clustering.</td></tr>
<tr><td>$(B_1, N, 3)$</td><td>$(B_1, N/4, 128)$</td><td>3D-GCN features</td></tr>
<tr><td>$(B_1, N/4, 128)$</td><td>$(B_1, 128)$</td><td>Max pooling</td></tr>
<tr><td>-</td><td>$(B_1, 128)$</td><td>Global features</td></tr>
</table>

				*Direction vectors and relative distances are internally computed by the 3D-GCN.
Aggregation layer	$(B_2, 35)$	-	Node features	B_2 denotes the total number of protein and ligand atoms across all samples in the current batch. B_2 is equal to the sum of $B_1^{\text{ligand}} + B_1^{pocket}$, which are the numbers of ligand and pocket atoms in the current batch.<br>35: including atomic symbol, degree, implicit valence, hybridization, and aromatic flag.
	$(B_2, 128)$	-	Point-cloud features	
	$(B_2, 35)$	$(B_2, 128)$	MLP	
	$[(B_2, 128), (B_2, 128)]$	$(B_2, 256)$	Concatenation	
	-	$(B_2, 256)$	Output	
HGCN (edge processing)	$(E_{ll}, 17)$	$(E_{ll}, 256)$	Intra-ligand edge mapping	E_{ll} : number of intra-ligand edges in the current batch.<br>E_{pp} : number of intra-pocket edges in the current batch.<br>E_{lp} : number of ligand→pocket inter-edges in the current batch.<br>E_{pl} : number of pocket→ligand inter-edges in the current batch.<br>11/17: inter-edge or intra-edge feature dimension, including bond-type indicators such as single, double, triple, aromatic, conjugated, and in-ring.
	$(E_{pp}, 17)$	$(E_{pp}, 256)$	Intra-pocket edge mapping	
	$(E_{lp}, 11)$	$(E_{lp}, 256)$	Ligand→pocket inter-edge mapping	
	$(E_{pl}, 11)$	$(E_{pl}, 256)$	Pocket→ligand inter-edge mapping	
HGCN (message passing)	Node:<br>$(N_{ligand}, 256)$<br>$(N_{pocket}, 256)$<br>Edge:<br>$(E_{ll}, 256)$,<br>$(E_{pp}, 256)$,<br>$(E_{lp}, 256)$,<br>$(E_{pl}, 256)$	-	Fused node features & Mapped edge features	N_{ligand} denotes the number of ligand atoms in the current batch, and N_{pocket} denotes the number of pocket atoms in the current batch. E_{ll}, E_{pp}, E_{lp}, and E_{pl} denote the numbers of edges of each edge type in the current batch.

	$(N_{ligand}, 256)$<br>$(N_{pocket}, 256)$<br>$(E_{ll}, 256)$,<br>$(E_{pp}, 256)$,<br>$(E_{lp}, 256)$,<br>$(E_{pl}, 256)$	$(N_{ligand}, 256)$,<br>$(N_{pocket}, 256)$,<br>$(E_{ll}, 256)$,<br>$(E_{pp}, 256)$,<br>$(E_{lp}, 256)$,<br>$(E_{pl}, 256)$	3-layer message passing	4 Convs:<br>CIGConv (intra_ligand): Sum<br>CIGConv (intra_pocket): Sum<br>NIGConv (inter_l2p): Mean<br>NIGConv (inter_p2l): Mean<br>Activation: LeakyReLU
	-	Node:<br>$(N_{ligand}, 256)$,<br>$(N_{pocket}, 256)$<br>Edge:<br>$(E_{ll}, 256)$,<br>$(E_{pp}, 256)$,<br>$(E_{lp}, 256)$,<br>$(E_{pl}, 256)$	Updated node and edge features	Used for affinity prediction
Output layer	Node:<br>$(N_{ligand}, 256)$<br>$(N_{pocket}, 256)$<br>Edge:<br>$(E_{lp}, 256)$	Edge-level score:<br>$(E_{lp}, 1)$<br>Global score:<br>$(1,)$	Ligand→pocket affinity (atompairs_lp)	Linear(256)_node +<br>Linear(256)_edge +<br>Interact(edge×src×dst) +<br>Linear(1)→EdgeScore $(E_{lp}, 1)$ +<br>Sum
	Node:<br>$(N_{ligand}, 256)$<br>$(N_{pocket}, 256)$<br>Edge:<br>$(E_{lp}, 256)$	$(1,)$	Ligand→pocket bias correction (bias_lp)	Linear(256)_node +<br>Linear(256)_edge +<br>Linear(1)+PReLU+Softmax +<br>(attn×edge×src×dst) + Sum + FC(1)
	$(1,),(1,)$	$(1,)$	Final ligand→pocket affinity	atompairs_lp - bias_lp
	Node:<br>$(N_{ligand}, 256)$<br>$(N_{pocket}, 256)$<br>Edge:<br>$(E_{pl}, 256)$	Edge-level score:<br>$(E_{pl}, 1)$<br>Global score:<br>$(1,)$	Pocket→ligand affinity (atompairs_pl)	Same as ligand→pocket

	Node: $(N_{ligand}, 256)$ $(N_{pocket}, 256)$ Edge: $(E_{pl}, 256)$	$(1,)$	Pocket→ligand bias correction (bias_pl)	Same as ligand→pocket
	$(1,),(1,)$	$(1,)$	Final pocket→ligand affinity	atompairs_pl - bias_pl
	Edge affinity: $(E_{lp}, 1)$, $(E_{pl}, 1)$	$(N_{ligand},)$	Ligand atom attention score	The edge-level affinity scores obtained from the two directions are aggregated to the ligand atoms using scatter_add_. Specifically, the ligand→pocket scores and the pocket→ligand scores are summed to produce a total_score, which is used to visualize the ligand atoms most emphasized by the model.
	$(1,),(1,)$	$(1,)$	Affinity prediction	(Pocket→ligand affinity + Ligand→pocke affinity) / 2

2 Dataset

2.1 The PDBbind Dataset

In this study, the primary dataset utilized for training and validation is derived from the PDBbind database, a comprehensive collection of experimentally measured binding affinity data for biomolecular complexes. To ensure data quality and compatibility with our processing pipeline, we performed a filtering step where samples that could not be successfully parsed by the RDKit toolkit were excluded. After this preprocessing, our final dataset consisted of a training set with 10,196 samples and a validation set with 1,149 samples. Within this dataset, the binding affinities are quantitatively expressed as their negative logarithmic values, specifically as pK_d (from dissociation constant K_d) or pK_i (from inhibition constant K_i). This is represented mathematically as $-\log K_d$ or $-\log K_i$, where a larger value signifies a stronger binding interaction between the protein and the ligand.

To rigorously evaluate the generalization capability of our model, we employed distinct and independent external test sets. In addition to standard benchmarks like the PDBbind 2013 core set ($N = 107$) and the 2016 core set ($N = 285$), we followed the methodology of Yang et al[1]. to establish a more realistic experimental setting using the PDBbind 2019 holdout set ($N = 4{,}366$). This configuration mimics a temporal split, where the model, trained on prior structural data, is used to predict binding affinities for newly released structures. Such a temporal evaluation not only poses a greater challenge but also more accurately simulates the real-world task of prospective drug discovery. Crucially, we have ensured that there is no overlap among the samples in the training, validation, and any of the three external test sets, guaranteeing an unbiased assessment of the model's performance on unseen data.

Furthermore, to assess our model's performance on in silico generated protein conformations, a scenario highly relevant to real-world applications where experimental structures may be unavailable, we conducted an additional evaluation. For the proteins in our test sets, we used AlphaFold2[2] to predict their three-dimensional structures from their primary amino acid sequences. These computationally predicted structures were then used as input for our model to predict binding affinities. This allowed us to evaluate the model's robustness to structural variations and to compare its performance against other state-of-the-art methods when operating on predicted, rather than experimentally determined, protein structures.

2.2 The DAVIS Dataset

The DAVIS dataset, introduced by Davis et al[3], is a pivotal resource for assessing kinase inhibitor selectivity. It provides comprehensive interaction data for 72 kinase inhibitors tested against a panel of 442 kinases, representing a significant portion of the human catalytic protein kinome. The dataset comprises 30,056 drug-protein interaction pairs, with each entry containing the drug's SMILES representation, the protein's amino acid sequence, and the corresponding binding affinity. For our analysis, the reported IC_{50} values were converted to their negative logarithmic form, pIC_{50}, to serve as the predictive target. To further benchmark the performance and robustness of our approach, we also evaluated our model on the DAVIS dataset. This allowed for a direct and rigorous comparison against other state-of-the-art models that have previously utilized this well-established dataset for performance validation.

2.3 The ZINC Dataset

To further explore the application of our methodology in a large-scale screening context, we utilized the ZINC database. ZINC is a free, curated collection of commercially available compounds specifically prepared for virtual screening and computational drug discovery[4]. For the purpose of our study, we downloaded a substantial subset of compounds from the ZINC database. This collection was subsequently employed as the chemical library for our molecular docking screening experiments, allowing us to identify potential hit compounds from a vast chemical space.

3 Performance Evaluation in a Cold Start Scenario

To comprehensively assess the model's predictive capabilities, we adopted four distinct data splitting strategies, as illustrated in Fig. 4i and Fig. 4j.

Warm-start (random split):

Random split is the most commonly used data-splitting strategy. Its advantages lie in its simplicity and in its ability to reflect training stability and average performance under an in-distribution setting. However, due to its randomness, homologous proteins or ligands with highly similar scaffolds may appear in both the training and test sets. This results in test data that are highly similar to the training cases, leading to implicit information leakage, or dataset bias, and tends to overestimate the model's true generalization capability.

Cold-start (sequence-identity/scaffold split/Complex-based split):

Following prior studies on cold-start data splitting strategies[1][5][6], we constructed out-of-distribution (OOD) data splits from both the protein and ligand perspectives to ensure that model evaluation involves previously unseen cases. The sequence-identity split is based on full-length protein sequence similarity. We use NW-align to compute pairwise sequence identities between

proteins and apply a 30% similarity threshold to prevent highly similar proteins from being split across different subsets. Under this split, proteins in the validation and test sets are considered sequence-dissimilar to those in the training set.

Correspondingly, the scaffold split constructs an OOD setting based on ligand scaffold similarity. Ligand structural similarity is quantified using Morgan fingerprint counts together with a set of physicochemical descriptors. We then apply threshold-based partitioning to ensure that ligands in the validation and test sets are structurally distinct from those in the training set. Under this split, validation and test ligands are considered dissimilar to the training ligands at the structural level, enabling a more realistic assessment of the model's generalization performance on novel-scaffold ligands.

The PLINDER-based complex split[7] constructs an OOD evaluation setting by partitioning protein-ligand complexes using a similarity graph defined by a chosen complex-level metric and threshold (e.g., protein sequence similarity or protein-ligand interaction similarity over aligned pocket residues). Test complexes are selected as a small number of representatives from clusters defined by a chosen similarity threshold, while controlling cluster size and limiting leakage by removing complexes connected to the selected test clusters. The remaining data are then split into training and validation using a similar partitioning procedure, yielding a validation set that is weakly connected to the training set under the configured similarity criterion.

4 Benchmarking Against State-of-the-Art Methods

Training on an NVIDIA A800 GPU takes 15 min 52 s per epoch on average, and early stopping is triggered after approximately 271 epochs (per-fold timings are provided in Supplementary Table 3). For inference on an NVIDIA RTX 4090, a single batch (batch size = 5) requires 1.1 GFLOPs and takes 1.7891 s.

Supplementary Table 3. Training details of E-CloudBind.

Fold	Early-stopping epochs	Training time (h)
1	258	61.78 h
2	274	91.95 h
3	288	68.69 h
4	328	78.33 h
5	265	73.77 h
6	327	89.88 h
7	291	55.03 h
8	202	56.17 h
9	178	48.87 h
10	260	61.68 h

To rigorously evaluate the performance of our proposed model, we conducted a comprehensive benchmarking analysis against several state-of-the-art methods for drug-target affinity prediction (comparative results are provided in **Supplementary Table 4**, with the detailed 10-fold cross-validation scatter distribution of our model shown in **Supplementary Figure 1**). The selected baselines span a diverse spectrum of architectural paradigms, including models that integrate both sequence-level and molecular graph-based information, those that rely solely on protein-ligand connectivity graphs, as well as advanced 3D graph neural networks capable of modeling fine-

grained geometric interactions. By benchmarking against such varied and competitive approaches, we provide a robust and objective assessment of our model's effectiveness and its standing in the current landscape of predictive modeling for molecular interactions.

- **DMFF** is a dual-modality neural network designed for drug-target affinity (DTA) prediction. The model integrates both sequence information and graph-based structural features for both drugs and proteins. Its core methodology relies on a binding site-focused graph construction approach to create an efficient representation of the drug-target interaction zone. We preprocessed PDBBind datasets and reproduced results using the open-source code from https://github.com/hehh77/DMFF-DTA.
- **PSICHIC** is a framework designed to predict protein-ligand interaction properties, including binding affinity and functional effects. Its defining characteristic is the ability to operate directly from sequence data alone (protein sequence and ligand SMILES), eliminating the need for high-resolution 3D structures. The model achieves this by incorporating physicochemical constraints into a graph neural network to decode interaction fingerprints. We preprocessed the relevant datasets and reproduced results using the open-source code from https://github.com/huankoh/PSICHIC.
- **SIGN** is a graph neural network designed to improve binding affinity prediction by more effectively using graph-level complex information. It addresses the limitations of standard GNNs by introducing two primary components: an intra-molecular graph convolution module and an inter-molecular graph convolution module. The intra-molecular module learns atom representations within the ligand and protein pocket, while the inter-molecular module captures pairwise atom-level interactions between the protein and ligand. Graph pooling is then used to aggregate the learned intermolecular edge representations for downstream affinity prediction. We preprocessed the relevant datasets and reproduced results using the open-source code from https://github.com/zjujdj/IGN.
- **EHIGN** is an explainable heterogeneous interaction graph neural network for protein-ligand binding affinity prediction that incorporates an interaction-based inductive bias from 3D complex structures. It represents protein-ligand complex as a heterogeneous graph that explicitly distinguishes covalent relations from non-covalent interactions, and predicts binding affinity by aggregating pairwise atom-atom interaction contributions derived from non-covalent edges, yielding an interpretable decomposition of the affinity prediction. We preprocessed the relevant datasets and reproduced the results using the authors' open-source implementation available at https://github.com/guaguabujianle/EHIGN_PLA.
- **FlowDock** is a geometric flow-matching model for generative protein-ligand docking and binding affinity prediction. Conditioned on unbound inputs, it learns to map structures to their bound counterparts, supports multi-ligand complex generation, and outputs predicted complex structures together with confidence estimates and affinity values. We preprocessed the relevant datasets and reproduced results using the open-source code from https://github.com/BioinfoMachineLearning/FlowDock.
- **Boltz-2** is a structural biology foundation model that achieves strong performance for both structure prediction and binding affinity prediction. It builds on the co-folding capabilities of its predecessor and is presented as advancing the frontiers of structure and affinity prediction. Architecturally, its co-folding trunk encodes inputs such as sequence, MSA, and templates into single and pair representations, and comprises an MSA module, a template module, and an

atom attention encoder, with a PairFormer backbone and recycling to iteratively refine representations and structure. The authors report that Boltz-2 approaches the performance of free-energy perturbation (FEP) methods for small-molecule-protein binding affinity estimation on their benchmarks while being at least 1000× more computationally efficient than FEP. We preprocessed the relevant datasets and reproduced results using the authors' open-source implementation at https://github.com/jwohlwend/boltz.

Supplementary Table 4. Comparison with State-of-the-Art Models.

Model	PDBBind 2013		PDBBind 2016		PDBBind 2019		Average	
	MAE	Pearson	MAE	Pearson	MAE	Pearson	MAE	Pearson
SIGN	1.0712	0.8154	**0.9002**	0.8369	1.1104	0.6385	1.0969	0.6545
DMFF	1.2039	0.7323	0.9602	0.8066	1.1907	0.5850	1.1788	0.6070
PSICHIC	1.2541	0.7150	1.0510	0.7706	1.1410	0.6108	1.1380	0.6281
FlowDock	1.7074	0.6435	1.5671	0.5332	1.6087	0.4872	1.3229	0.4935
EHIGN	1.1973	0.7210	1.1506	0.7449	1.2741	0.6571	1.2649	0.6638
Boltz-2	1.2656	0.7652	1.2240	0.7409	1.1512	0.5768	1.1679	0.5936
Ours	**1.0391**	**0.8334**	0.9245	**0.8466**	**1.0683**	**0.6628**	**1.0590**	**0.6672**

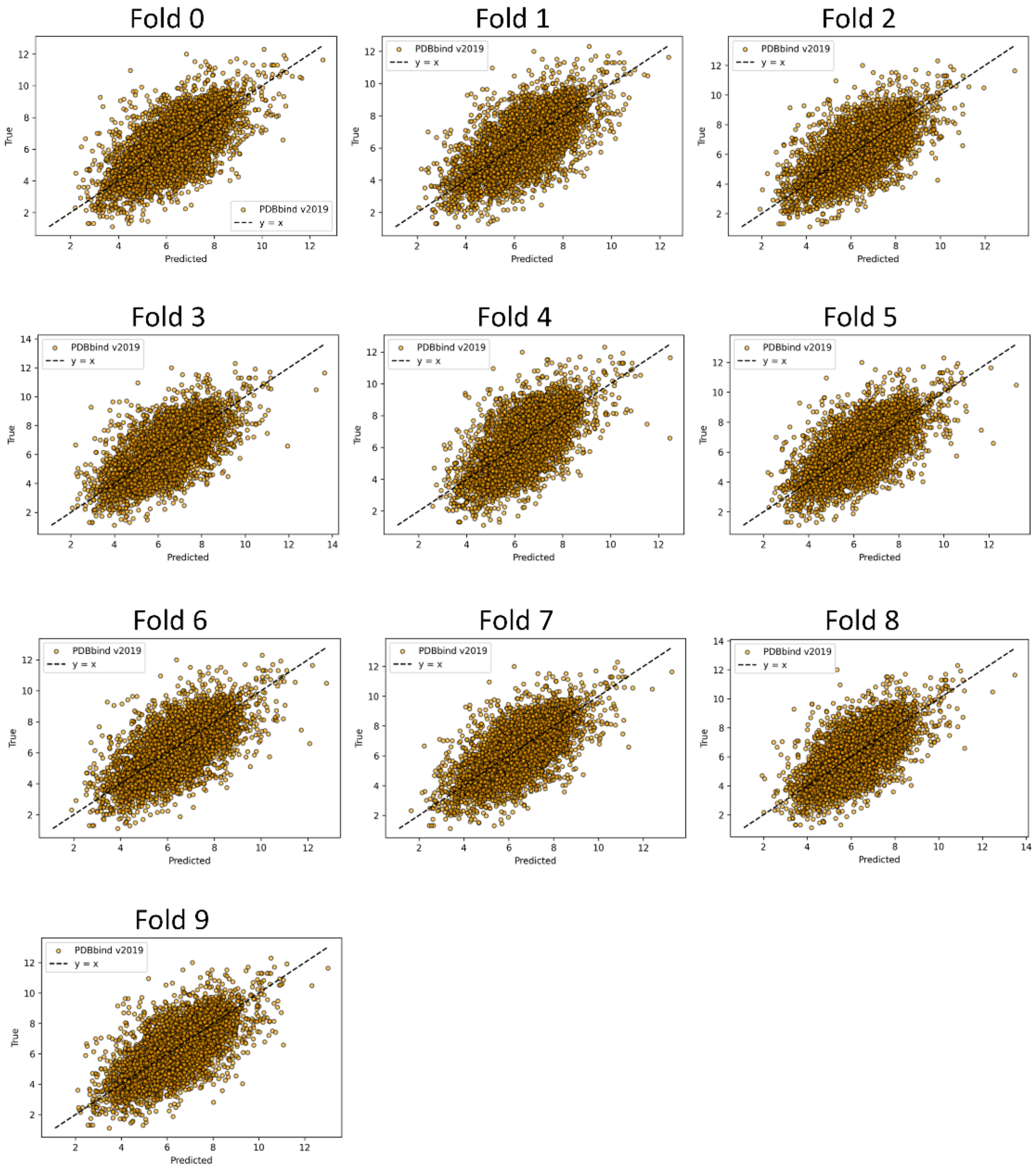

Supplementary Figure 1. Prediction performance of the model on the PDBbind v2019 test set using 10-fold cross-validation. Each subplot represents the results for a specific fold, showing the scatter plot between predicted and true values. The results demonstrate stable and excellent performance across all folds. Among all folds, the best MAE achieved on the test set was **1.060** and the best Pearson correlation coefficient was **0.672**. These results further confirm the effectiveness and robustness of the model in molecular binding prediction tasks.

5 Sampling Based on van der Waals Radii

To investigate the probabilistic spatial distribution around atoms, we performed multivariate Gaussian sampling for six representative atom types within a sphere defined by two times the van der Waals (vdW) radius. As shown in **Supplementary Figure 2**, redder regions in the density map indicate areas of higher sampling probability. The results demonstrate a strong enrichment of sampling points within the inner region (i.e., within 1 × vdW radius), suggesting a significantly higher spatial occupancy in the tightly constrained domain compared to the outer, more loosely defined region (up to 2 × vdW radius).

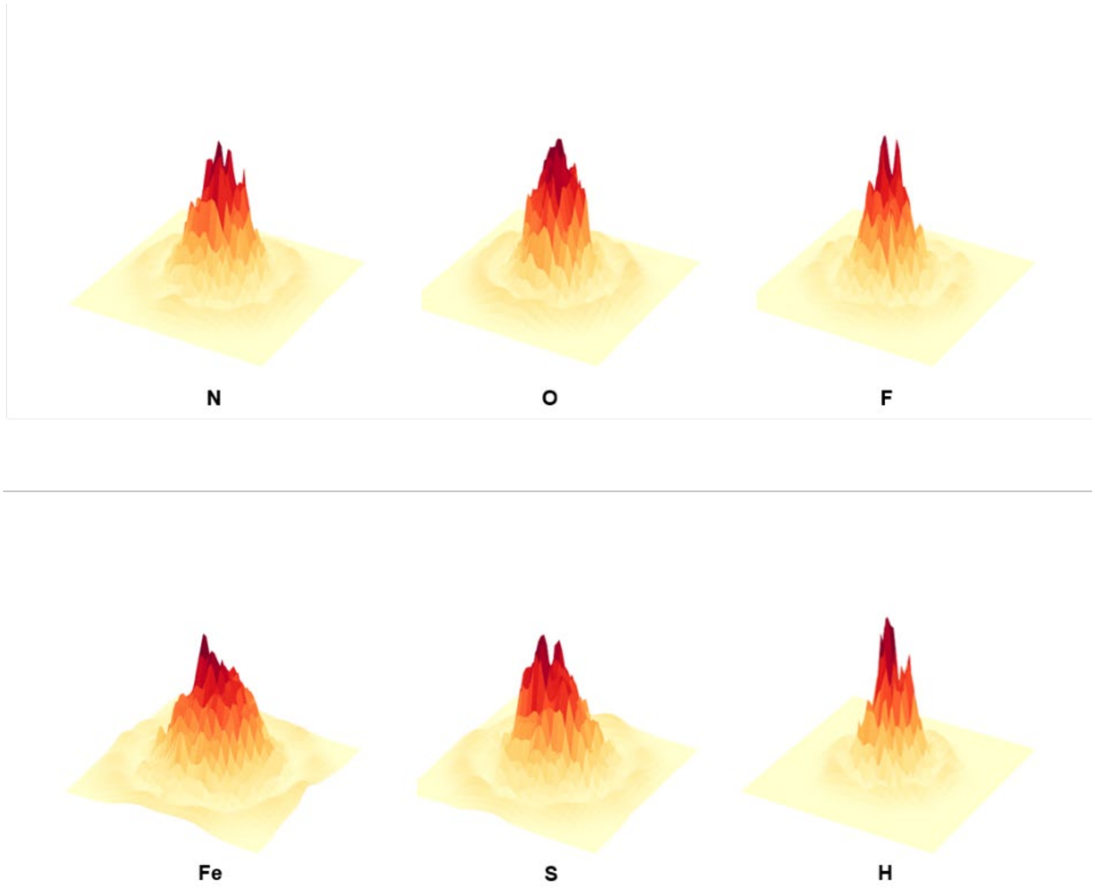

Supplementary Figure 2. Sampled electron density distribution within two times the van der Waals radius for six representative atoms. Redder regions indicate higher sampling probability. A clear enrichment is observed in the tightly constrained region (within 1× van der Waals radius), where the number of samples significantly exceeds that in the loosely constrained outer region (within 2× radius).

6 Electron-like Sampling Strategy

The fuzzification of protein structures aims to capture spatial uncertainty by generating a cloud of sampled points around each atom. This is achieved by applying multivariate Gaussian sampling centered at the actual atomic coordinates, with an inner and outer constraint defined by 1× and 2× van der Waals radii, respectively. As illustrated in **Supplementary Figure 3**, the top schematic outlines the basic sampling strategy, while the bottom panel presents the 3D visualization of fuzzified point clouds for protein pockets from four distinct PDB entries: 1Q8T, 2XBV, 3AO4, and 4TY7. The resulting distributions reflect both the geometry and the variability introduced by the fuzzy representation.

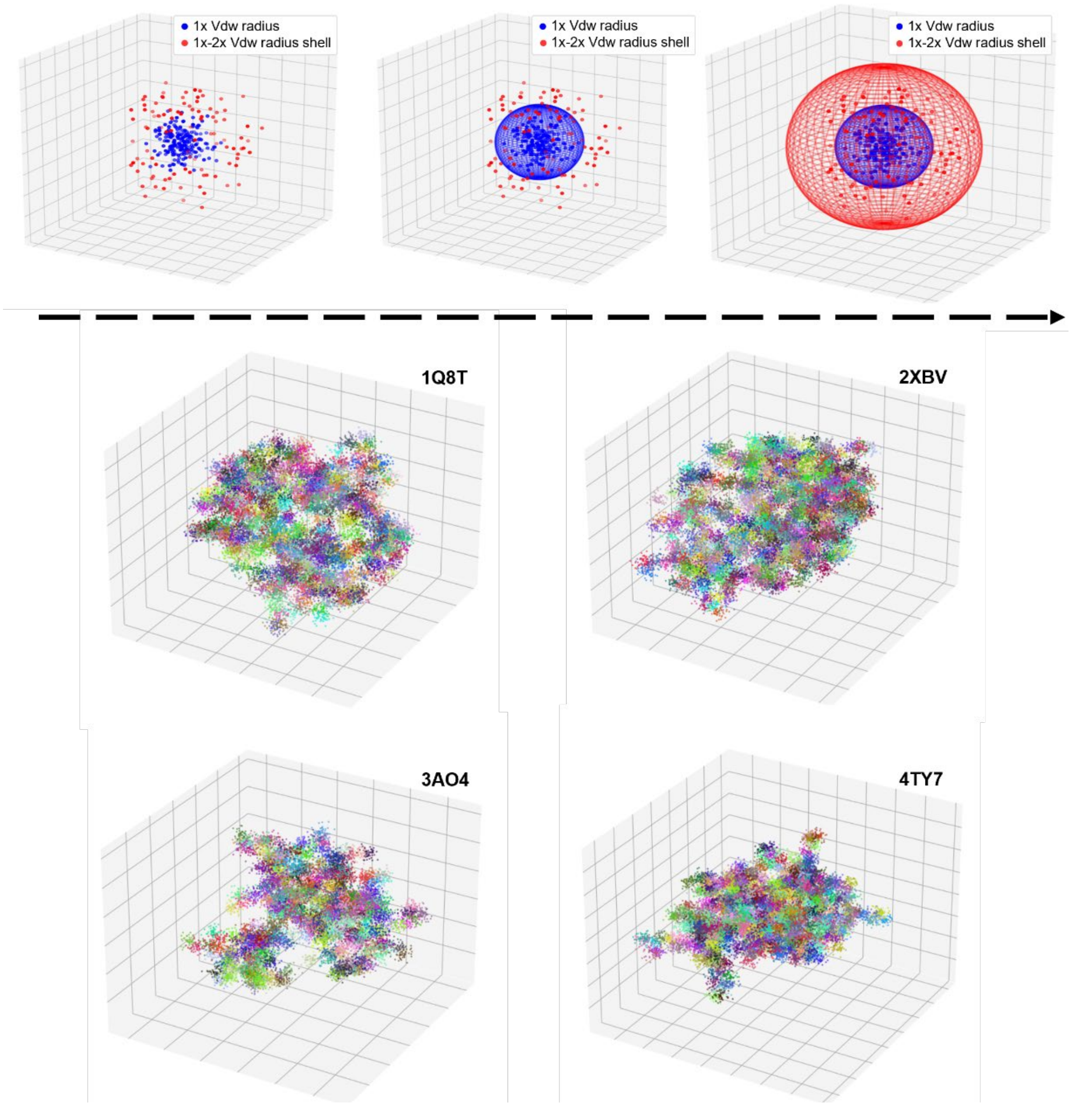

Supplementary Figure 3. The basic idea of fuzzifying protein structures is to perform multivariate Gaussian sampling using each atom's actual coordinate as the center, with an inner sphere defined by one van der Waals radius and an outer sphere defined by two van der Waals radii. The upper figure illustrates this conceptual framework, while the lower figure shows the 3D visualization of fuzzified sampling for four protein pockets (PDB IDs: 1Q8T, 2XBV, 3AO4, and 4TY7).

7 Case Study

To understand the model's attention to atomic-level interactions, we visualized the predicted noncovalent contacts for ligands bound to the protein pockets of PDB IDs 2FDD and 2ZJV. As shown in **Supplementary Figure 4**, attention scores at the atom level are overlaid onto the ligands, with darker red tones representing stronger model attention. Key noncovalent interactions, particularly hydrogen bonds, are indicated by dashed green lines. The protein pockets are rendered in transparent cartoon mode to provide a clear view of the binding geometry and highlight the localized regions where the model focuses its interpretive capacity.

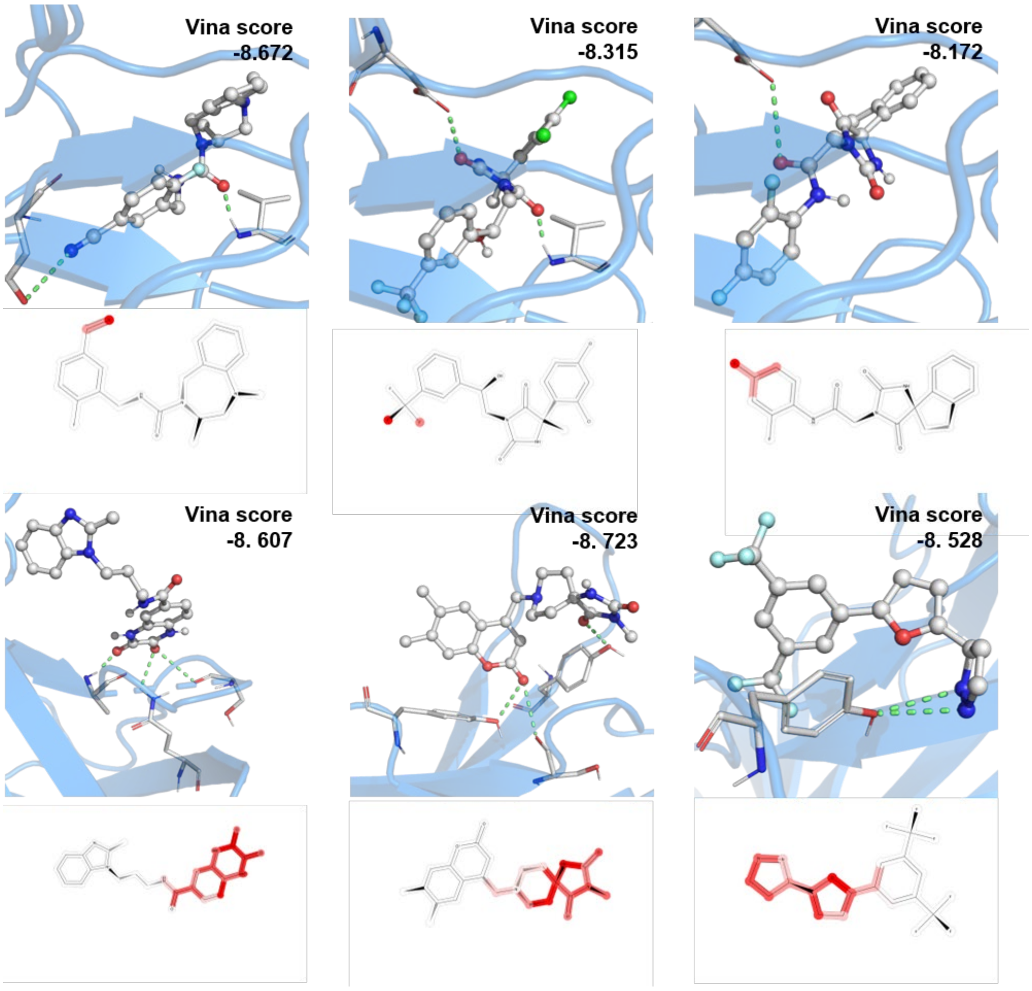

Supplementary Figure 4. Visualization of noncovalent interactions for representative ligands targeting protein pockets from PDB IDs 2FDD and 2ZJV. Atom-level attention scores are mapped onto the ligands, where darker red corresponds to higher model attention. Key noncovalent contacts, such as hydrogen bonds, are highlighted with dashed green lines. Protein pockets are shown in transparent cartoon mode for clarity.

Reference

[1] Yang Z, Zhong W, Lv Q, et al. Interaction-based inductive bias in graph neural networks: enhancing protein-ligand binding affinity predictions from 3d structures[J]. IEEE Transactions on Pattern Analysis and Machine Intelligence, 2024.

[2] Jumper J, Evans R, Pritzel A, et al. Highly accurate protein structure prediction with AlphaFold[J]. Nature, 2021, 596(7873): 583-589.

[3] Davis M I, Hunt J P, Herrgard S, et al. Comprehensive analysis of kinase inhibitor selectivity[J]. Nature Biotechnology, 2011, 29(11): 1046-1051.

[4] Sterling T, Irwin J J. ZINC 15-ligand discovery for everyone[J]. Journal of Chemical Information and Modeling, 2015, 55(11): 2324-2337.

[5] Luo Y, Liu Y, Peng J. Calibrated geometric deep learning improves kinase-drug binding predictions[J]. Nature Machine Intelligence, 2023, 5(12): 1390-1401.

[6] Li H, Peng J, Sidorov P, et al. Classical scoring functions for docking are unable to exploit large volumes of structural and interaction data[J]. Bioinformatics, 2019, 35(20): 3989-3995.

[7] Durairaj J, Adeshina Y, Cao Z, et al. PLINDER: the protein-ligand interactions dataset and evaluation resource[J]. BioRxiv, 2024: 2024.07. 17.603955.
