# Supplementary material for: An electron-density point-cloud framework for robust protein-ligand interaction prediction
Source: Nat Commun. 2026 Jun 11;17:7424. doi: 10.1038/s41467-026-74196-5 (PMC13408660; doi:10.1038/s41467-026-74196-5)
Supplement: Supplementary file 2 — Description of Additional Supplementary Files [file 41467_2026_74196_MOESM2_ESM.pdf]

Description of Additional Supplementary Files

Supplementary Data 1: Supplementary plotting files for all figures, including files in PZFX and PSE formats.
