# Supplementary material for: An electron-density point-cloud framework for robust protein-ligand interaction prediction
Source: Nat Commun. 2026 Jun 11;17:7424. doi: 10.1038/s41467-026-74196-5 (PMC13408660; doi:10.1038/s41467-026-74196-5)
Supplement: Supplementary file 4 — Transparent Peer Review file [file 41467_2026_74196_MOESM4_ESM.pdf]

nature portfolio

Peer Review File

An Electron-Density Point-Cloud Framework for Robust Protein-Ligand Interaction Prediction

Corresponding Author: Professor Shidang Xu

This file contains all reviewer reports in order by version, followed by all author rebuttals in order by version.

Version 0:

Reviewer comments:

Reviewer #1

(Remarks to the Author)
Review for Liu, Wang, and Wang et al: This manuscript presents a new framework for predicting non-covalent protein-ligand interactions by representing the complexes using quantum mechanical electron density clouds. In doing so, the predicted interactions are based on real physical principles instead of using simple distance cutoffs. It provides a more accurate description of non-covalent interactions and the authors highlight an advantage is that their approach can handle differences in structural resolution that can arise from varying experimental quality. Overall, the method is presented well and is technically very interesting.

However, many computational studies lack experimental validation (including this work) which would greatly strengthen the practical relevance of the work. Could the authors test 1-2 predicted compounds from Figure 6 for experimental target binding, especially for a system like BCL-2 where the screened compound is predicted to be much more favorable than navitoclax? The authors used the ZINC database, notable for its inclusion of compounds with synthetic feasibility and commercial availability. If experimental validation is not possible, can the authors evaluate and report the synthetic accessibility of the predicted compounds?

Other minor comments:
The authors use the word “significant” several times in the discussion of figure 3: “Specifically, we compared with state-of-art methods for different types of inputs and achieved significantly leading performance.” And “All baseline models demonstrated a significant increase in prediction error as structural resolution declined (i.e., higher Å values), with regression slopes of 0.065, 0.060, and 0.053, respectively” While the performance metrics for ECloudbind are better, they are only marginally better than the baseline models. Are these differences actually statistically significant? If not, please change the language in the text to reflect this.

In the caption of Figure 1: panel a is described “Since the same radius is applied to all elements, atom identity is ignored, leading to spurious contacts in some cases (red arrow).” Please correct red arrow to red dashed line for clarity.

(Remarks on code availability)

Reviewer #2

(Remarks to the Author)
Summary: The authors introduce E-CloudBind, a robust graph-based method for protein-ligand binding affinity prediction. Empirical results across PDBBind-based data splits suggest the utility of the authors' proposed method. Nonetheless, significant concerns remain regarding data leakage and the omission of important recent baseline methods.

Points of strength:
1. The authors' proposed method, E-CloudBind, is well-motivated from the perspective of robustly parametrizing protein-ligand binding structures in light of noisy or inaccurate input structures.
2. Empirical results support the idea that E-CloudBind works well with both predicted and experimental protein structures for

protein-ligand binding affinity prediction.

Points for improvement:
1. My main concern is that the authors' benchmarking datasets (PDBBind 2013-2019) are not distinguished from their method's training dataset in a rigorous manner for protein-ligand interaction modeling. Previous benchmarks such as PLINDER [1] demonstrated the importance of partitioning protein-ligand interaction data according to strict (joint) protein-ligand binding similarity measures. However, the authors currently remove redundancy according to protein or ligand similarity but not both concurrently.
2. Another significant concern is that the authors have omitted two important baseline methods for comparison: Boltz-2 for biomolecular structure and affinity prediction [2] and FlowDock for protein-ligand docking and affinity prediction [3]. These methods have previously been benchmarked using CASP16 and PDB-wide evaluation data. To address my primary concerns for this manuscript, I would recommend that the authors compare their method to (at least) Boltz-2 for affinity prediction using (as close to) their experimental setup (i.e., training, validation, and testing data and evaluation metrics) as possible.

References:
1. Durairaj, J., Adeshina, Y., Cao, Z., Zhang, X., Oleinikovas, V., Duignan, T., ... & Naef, L. PLINDER: The protein-ligand interactions dataset and evaluation resource. In ICML'24 Workshop ML for Life and Material Science: From Theory to Industry Applications.
2. Passaro, S., Corso, G., Wohlwend, J., Reveiz, M., Thaler, S., Somnath, V. R., ... & Barzilay, R. (2025). Boltz-2: Towards accurate and efficient binding affinity prediction. BioRxiv.
3. Alex Morehead, Jianlin Cheng, FlowDock: Geometric flow matching for generative protein–ligand docking and affinity prediction, Bioinformatics, Volume 41, Issue Supplement_1, July 2025, Pages i198–i206, https://doi.org/10.1093/bioinformatics/btaf187

(Remarks on code availability)
I believe the authors' provided code and documentation is sufficient for reproducing the results of this paper.

Reviewer #3

(Remarks to the Author)
The manuscript "An Electron-Density Point-Cloud Framework for Robust Protein-Ligand Interaction Prediction" by Professor Xu and colleagues introduces a new machine learning framework for exploiting electron-density-based representations, combined with more traditional atomic structure representations, for the prediction of protein-ligand binding affinities.

The paper presents a number of relevant novel ideas and concepts, and might be suitable for publication in Nature Communications once the following points are addressed:

- Some similar/related work is not mentioned and discussing similarities and differences would be beneficial to provide the reader with relevant context: https://arxiv.org/html/2505.19014v1, https://pubs.rsc.org/en/content/articlehtml/2024/ra/d3ra08650, potentially older work on QTAIM by Bader et al.

- "E-CloudBind employs Gaussian sampling within adaptive envelopes (bottom) to generate a point cloud representation that is inherently rotation- and translation-invariant" → please explain more in detail. Do you mean rotation/translations to the entire structure? If so, the same argument holds for purely distance-based methods.

- Additional details on how electron-density based features are assigned to individual atoms following k-means clustering would be useful.

- "The specific running results are in the supplementary information." → Please point to the specific section/table in the supporting information.

- Please clarify wether the models you're choosing as a comparison (SIGN, PSICHIC, and DMFF) have been trained on the same data splits as your model.

- Please comment on the choice of dataset split (random/structural/temporal) and on the benefits/shortcomings of the chosen strategy.

- "[In order to] quantify robustness to crystallographic resolution, atomic coordinates were progressively perturbed to mimic structures solved at 1.9, 2.7 and 3.4 Å" → I understand you've added random coordinate noise, is this correct? If so, this would severely distort the underlying structures, even though also low-resolution structures from crystallographic or cryo-EM experiments would show (mostly) internally-constistent bond-length/angles etc. As an alternative experiment, I would suggest not adding coordinate noise, but rather stratifying the results on a hold-out test set by the respective resolutions of the experiments, i.e. what's the error on structures with resolution < 1.9 A, 1.9 A <= resolution < 2.7 A etc.

- In Figure 2a, you're mentioning frontier orbital calculations, though my understanding from the remainder of the paper is that you're not limiting your analysis to frontier orbitals, but are rather including the entire electron density. It would be good to clarify this.

- Fig 3c: bar plots with vertical bars would be easier to read and would be in line with Fig 3d. Please also ensure same ordering of methods for ease of comparison across subplots.

- The axis labelling in Figure 3e could be improved. It's not quite clear to me what you mean by "complexity-condited heat map" (i.e., how is ligand or protein complexity measured? - there are many ways to do this). What does "better complexity" mean? Higher or lower?

- Figure 3d: the AE distribution of "Ours" does not seem to correspond to median error of 0.870.

- "p-value as high as 0.703" → please clearly state the statistical test you're using here.

- "negligible reduction in Pearson of 0.004" → "negligible reduction in Pearson correlation coefficient..." would be more correct (same in other sentences)

- Figure 3 vs Figure 4 → why do you switch the metric from MAE to RMSE?; same from Figure 4b <> 4d.

- Figure 4: since you're discussing speed-ups: could you describe the computation cost/walltime requirements for computing electron densities/feature preparation/model training/inference time? GFN2-xTB is quite fast, particularly for small ligands.

- Figure 4 shows that "the inexpensive Gaussian procedure matches the quantum-derived variant" - doesn't this contradict some of the other points you've made in the paper, i.e. that electron density is a useful description to assess protein-ligand binding affinity?

- Fig. 4d, MAE plot: bar plots coming from the top are very confusing and I've never seen one.

- Fig 5: color bars are confusing because the label "0 → 1" with the arrow pointing downwards implies that lighter green means 1, and darker green means 0, though the opposite is the case. Please also show the colors for all other atoms, including carbon atoms, for better comparability. Please clearly explain which attention score (from which part of the model) you're reporting.

- "Four representative protein-ligand complexes were selected (PDBs: 1BCU, 3PXF, 5WMT, and 6DAR)". Please explain your motivation for choosing these specific complexes. Also, you are not citing papers for any of these PDBs. Please do so.

- Question on virtual screening: "The top 5% of candidates, ranked by the model's predicted affinity scores, were subjected to molecular docking for validation." Do I understand correctly that since the model separately aggregates protein and ligand sub-graphs, the relative orientation of the two is not taken into account, and so (e.g. for virtual screening), you don't need the full complex geometry, but just a conformation for the ligand and the protein separately? If so, one could argue that for structure-based approaches, you want to negatively score wrongly oriented poses - or is the argument that the network implicitly reasons over orientation?

- You've shown that your model is resistant to coordinate noise, and I'd be curious to see a negative control:At which point does the model break down? Clearly if you distort the structures too much, you wouldn't want your model to output a positive binding score, because it would be based on entirely incorrect geometries that would never bind. E.g. rotate the ligand in a non-binding pose (that would depend on the answers to the previous question), distort the ligand which would mean large internal strain, ...

- Additional details on the network architecture (e.g. in a table) would be appreciated, i.e. number of message passing aggregations, specific atom/bond features etc. Which features from the electron density are actually going into the network?

- Could you motivate your choice of global max-pooling over the downsampled ligand and protein features? Doesn't this mean you only take into account one atom from the ligand and one atom from the protein or are you max-pooling over a different dimension?

(Remarks on code availability)

Version 1:

Reviewer comments:

Reviewer #1

(Remarks to the Author)
The authors have responded to my comments and revised the manuscript accordingly. I'm satisfied with the responses and have no further comments or queries.

(Remarks on code availability)
I have not run the code.

Reviewer #2

(Remarks to the Author)
I would like to thank the authors for their detailed rebuttals. Overall, their responses have addressed my main concerns, and I am happy to see this manuscript published in its current form.

(Remarks on code availability)
The authors' code appears to be well-documented and structured.

Reviewer #3

(Remarks to the Author)
Thank you for addressing the points I've raised during the review process. The questions and suggestions I made were all addressed, and I recommend publication of this manuscript.

(Remarks on code availability)

Open Access This Peer Review File is licensed under a Creative Commons Attribution 4.0 International License, which permits use, sharing, adaptation, distribution and reproduction in any medium or format, as long as you give appropriate credit to the original author(s) and the source, provide a link to the Creative Commons license, and indicate if changes were made.
In cases where reviewers are anonymous, credit should be given to 'Anonymous Referee' and the source.
The images or other third party material in this Peer Review File are included in the article’s Creative Commons license, unless indicated otherwise in a credit line to the material. If material is not included in the article’s Creative Commons license and your intended use is not permitted by statutory regulation or exceeds the permitted use, you will need to obtain permission directly from the copyright holder.
To view a copy of this license, visit https://creativecommons.org/licenses/by/4.0/

Catalog

Response to Reviewers' Comments 2
To Reviewer #1 3
To Reviewer #2 6
To Reviewer #3 8
Reference 22

Response to Reviewers' Comments

Dear Reviewers, thank you very much for your comments regarding on our manuscript. We are truly grateful to your thoughtful suggestions. Your insightful feedback has significantly helped us improve both the preciseness and practical significance of the study.

The comments from Reviewer #1 encouraged us to extend our analysis beyond the existing virtual screening results and conduct more in-depth investigations. Following this suggestion, we conducted additional experiments and analyses to enhance the real-world relevance and practical value of our work.

We are also grateful to Reviewer #2 for the important suggestion to introduce a new data split. In response, we incorporated this new complex-based split setting to establish a more rigorous cold-start evaluation protocol. This modification strengthens the experimental design and provides a more rigorous assessment of the E-CloudBind's generalization capability.

Reviewer #3's valuable comments motivated us to conduct a more comprehensive exploration of the effects of structural rotations and translations on E-CloudBind performance. We evaluated the robustness of E-CloudBind under these geometric transformations. In addition, we have carefully revised and supplemented methodological and implementation details throughout the manuscript, further improving its clarity, rigor, and overall persuasiveness.

We have highlighted all revisions in the revised manuscript in yellow and we believe that the revised manuscript has been strengthened as a result of your thoughtful guidance, and we sincerely thank you again for your constructive feedback.

To Reviewer #1

Reviewer #1, Question 1: *many computational studies lack experimental validation (including this work) which would greatly strengthen the practical relevance of the work. Could the authors test 1-2 predicted compounds from Figure 6 for experimental target binding, especially for a system like BCL-2 where the screened compound is predicted to be much more favorable than navitoclax? The authors used the ZINC database, notable for its inclusion of compounds with synthetic feasibility and commercial availability. If experimental validation is not possible, can the authors evaluate and report the synthetic accessibility of the predicted compounds?*

Response: Thank you for raising this important point regarding experimental validation and the practical feasibility of the screened compounds. While we currently do not have access to the required wet-lab facilities to perform target-binding assays, we have strengthened the manuscript by providing additional evidence on (i) the synthetic feasibility of the top-ranked candidates and (ii) their binding stability under molecular dynamics (MD) simulations for the BCL-2 system.

Specifically, we conducted virtual screening on the ZINC database using BCL-2[1][2] as the target receptor, and evaluated the top 10,000 candidates with two synthesizability metrics, SAScore (Synthetic Accessibility Score, SAScore)[3] and SCScore (Synthetic Complexity Score)[4]. SAScore ranges from 1 to 10 (higher values indicate harder synthesis), while SCScore ranges from 1 to 5 (higher values indicate greater synthetic complexity). Following prior benchmarks[5][6], we use empirical cutoffs of SAScore = 6.0 and SCScore = 4, where SAScore $\leq$ 6.0 and SCScore $\leq$ 4 indicate reasonable synthesizability/acceptable synthetic complexity, and higher values suggest increasingly challenging synthesis. Under these criteria, among the top 10,000 candidates, 99.77% (9977/10,000) satisfy SAScore $\leq$ 6.0, 74.34% (7434/10,000) satisfy SCScore $\leq$ 4, and 74.17% (7417/10,000) satisfy both thresholds simultaneously. The overall distributions of these two metrics are shown in Fig. 7a, indicating that the screened candidates generally fall within a reasonable range of synthesizability/complexity and are not noticeably biased toward extremely difficult-to-synthesize compounds with high SAScore or SCScore.

In addition, we performed MD simulations to further assess the binding stability of candidates for BCL-2 (Fig. 7b and c). MD simulations are widely used as a computational validation to probe whether predicted ligands can maintain stable pocket interactions under realistic thermal fluctuations[7][8][9]. We selected the same screened compounds as in Fig. 6 (ZINC38931626, ZINC94753678, and ZINC57101123), together with an experimentally validated reference ligand (navitoclax) and simulated

the corresponding protein-ligand complexes under consistent conditions (400 ns per complex, explicit solvent with the TIP3P water model, and the AMBER14SB force field), with full details provided in the *Molecular Dynamics Simulations* section (lines 714-735) of the revised manuscript. Ligand RMSD was monitored over time after alignment to the protein backbone to quantify conformational stability within the binding pocket (Fig. 7b). The candidates exhibit stable RMSD trajectories without abrupt drift indicative of dissociation, and their RMSD profiles are comparable to those of the reference ligand over the simulation window. We further provide representative bound conformations (Fig. 7c) and estimate binding strengths using trajectory-based binding free-energy calculations (reported as "Binding Affinity", kcal·mol^{-1}; method: gmx mmpbsa). The candidates show favorable binding affinity estimates, which are consistent with stable and physically plausible binding in the BCL-2.

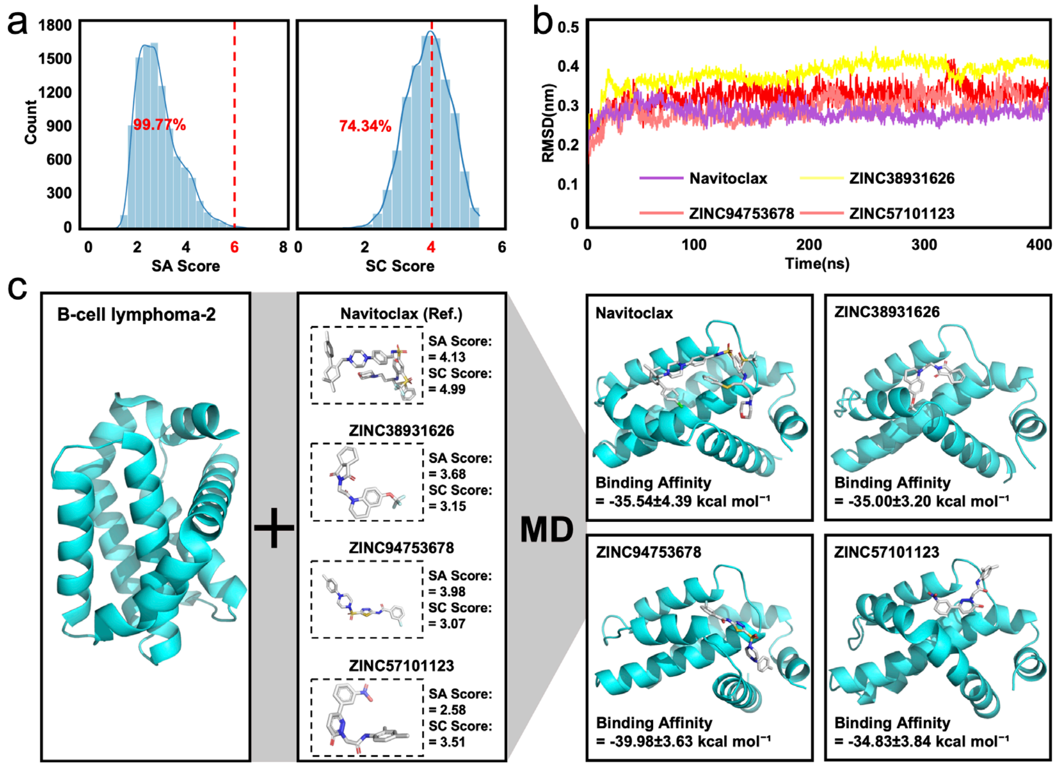

Figure 7 | Virtual Screening Analysis and Molecular Dynamics Simulation Results. a. Distributions of synthesizability metrics (SAScore and SCScore). For the top 10,000 ZINC candidates, empirical cutoffs (SAScore ≤ 6.0; SCScore ≤ 4.0) yield pass rates of 99.77%, 74.34%, and 74.17% for SAScore, SCScore, and both, respectively. **b. RMSD analysis of the reference ligand (Navitoclax), ZINC38931626, ZINC94753678 and ZINC57101123 complexes.** RMSD was plotted over 400 ns for Navitoclax and candidates. In all systems, ligand RMSD reaches a plateau after 100 ns and remains bounded within 0.20-0.35 nm, with no evidence of abrupt drift. **c. Representative Molecular Dynamics (MD) simulation results and corresponding binding affinities.** MD simulations were performed with GROMACS, and the resulting trajectories were

used to estimate binding affinities (kcal·mol⁻¹) for the candidates. The candidates exhibit affinities comparable to the reference ligand, consistent with stable binding in the BCL-2 pocket.

Reviewer #1, Question 2: *The authors use the word "significant" several times in the discussion of figure 3: "Specifically, we compared with state-of-art methods for different types of inputs and achieved significantly leading performance." And "All baseline models demonstrated a significant increase in prediction error as structural resolution declined (i.e., higher Å values), with regression slopes of 0.065, 0.060, and 0.053, respectively" While the performance metrics for E-Cloudbind are better, they are only marginally better than the baseline models. Are these differences actually statistically significant? If not, please change the language in the text to reflect this.*

Response: Thank you for your advice on improving the rigor of our manuscript statements. We re-checked the manuscript and removed inappropriate uses of "significant" in cases where statistical significance was not established or where performance differences were small.

We removed "significantly" from the sentence: "Specifically, we compared with state-of-the-art methods for different types of inputs and achieved ~~significantly~~ superior performance, as detailed in Supplementary Table 4." In addition, we revised the sentence: "All baseline models demonstrated a significant increase in prediction error as structural resolution declined (i.e., higher Å values), with regression slopes of 0.065, 0.060, and 0.053, respectively." as follows: "All baseline models exhibited a consistent increase in prediction error as structural resolution declined (i.e., higher Å values), with regression slopes of 0.065, 0.060, and 0.053, respectively."

Reviewer #1, Question 3: *In the caption of Figure 1: panel a is described "Since the same radius is applied to all elements, atom identity is ignored, leading to spurious contacts in some cases (red arrow)." Please correct red arrow to red dashed line for clarity.*

Response: Thank you for pointing out the error in the manuscript. In the caption of Fig. 1, we have corrected "red arrow" to "red dashed line" for clarity in the revised manuscript. The revised caption for Fig. 1a is:

Conventional DPI models adopt a uniform cutoff scheme, defining a non-covalent interaction (NCI) whenever any receptor and ligand atoms fall within a global Euclidean cutoff (e.g., 2.5 Å; yellow envelopes). Because the same radius is applied to all elements, atom identity is ignored, which can lead to spurious contacts in some cases (red dashed line). The resulting interaction graph is then fed into a graph neural network (GNN) for affinity prediction via message passing.

To Reviewer #2

Reviewer #2, Question 1: *My main concern is that the authors' benchmarking datasets (PDBBind 2013-2019) are not distinguished from their method's training dataset in a rigorous manner for protein-ligand interaction modeling. Previous benchmarks such as PLINDER demonstrated the importance of partitioning protein-ligand interaction data according to strict (joint) protein-ligand binding similarity measures. However, the authors currently remove redundancy according to protein or ligand similarity but not both concurrently.*

Response: Thank you for the advice, and we agree with your concern. In addition to the cold-start evaluations conducted separately on the ligand side (scaffold split) and the protein side (sequence-identity split), we have now incorporated a complex-level partitioning protocol based on PLINDER[10], which jointly accounts for protein-ligand binding similarity. The results for this PLINDER-based split are reported in Fig. 4h-j, with additional description and discussion provided in the Supplementary Information (*Performance Evaluation in a Cold Start Scenario* section) and in the third paragraph of the *Ablation and Generalization Analysis* section (lines 324-340). The revised text is provided below:

To further examine its generalization capabilities under shifts in training data distribution, two evaluation paradigms were employed: transductive (warm-start) and inductive (cold-start) settings. Specifically, the transductive setting involved random partitioning of the dataset into training and testing subsets. The inductive setting introduced a greater distributional divergence by holding out training and testing splits based on (i) protein sequence similarity, (ii) ligand scaffolds, and (iii) joint protein-ligand complex similarity, ensuring evaluation on previously unseen patterns (Fig. 4h). As shown in Fig. 4i, E-CloudBind consistently achieved superior generalization performance across both settings, yielding the lowest MAE (0.811) and highest Pearson correlation coefficient (0.830), with minimal error fluctuations of 0.009 and 0.00126, respectively. In contrast, baseline models exhibited clear performance degradation under the inductive condition, marked by a notable increase in MAE and decline in Pearson. Furthermore, Fig. 4j illustrates that E-CloudBind maintained stable prediction accuracy across protein structures with varying resolution, regardless of transductive or inductive partitioning. No significant performance drops were observed due to structural perturbations or partition-induced shifts. In comparison, models such as SIGN, PSICHIC, and DMFF demonstrated a clear trend of increasing prediction error

as structural resolution declined, reinforcing the broader applicability and robustness of E-CloudBind in practical deployment scenarios.

***Reviewer #2, Question 2:** Another significant concern is that the authors have omitted two important baseline methods for comparison: Boltz-2 for biomolecular structure and affinity prediction and FlowDock for protein-ligand docking and affinity prediction. These methods have previously been benchmarked using CASP16 and PDB-wide evaluation data. To address my primary concerns for this manuscript, I would recommend that the authors compare their method to (at least) Boltz-2 for affinity prediction using (as close to) their experimental setup (i.e., training, validation, and testing data and evaluation metrics) as possible.*

Response: Thank you for the advice, and we agree that Boltz-2[11] and FlowDock[12] are important baselines. We have now evaluated both methods under the exact same software, hardware, and data split used for E-CloudBind. For Boltz-2, we performed inference-only evaluation on our test set using the official pretrained weights and default hyperparameter settings, since the training code is not publicly available. For FlowDock, we used the official training code with the default hyperparameters, and trained and evaluated the model on the same train/validation/test split used for E-CloudBind. The results for mean absolute error (MAE) and Pearson correlation coefficient (Pearson) are summarized in Table 1 (or Supplementary Table 4) and discussed in the revised manuscript in the *Robustness Analysis Across Heterogeneous Structural Quality* section (lines 165-168), with additional details in the Supplementary Information (*Benchmarking Against State-of-the-Art Methods*). E-CloudBind consistently outperforms all baselines across the three datasets. To ensure transparency and reproducibility, we also provide detailed prediction outputs for both baselines in the supplementary files for reference.

Table 1. Comparison with State-of-the-Art Models.

Model	PDBBind 2013		PDBBind 2016		PDBBind 2019		Average	
	**MAE**	**Pearson**	**MAE**	**Pearson**	**MAE**	**Pearson**	**MAE**	**Pearson**
SIGN	1.0712	0.8154	0.9002	0.8369	1.1104	0.6385	1.0969	0.6545
DMFF	1.2039	0.7323	0.9602	0.8066	1.1907	0.5850	1.1788	0.6070
PSICHIC	1.2541	0.7150	1.0510	0.7706	1.1410	0.6108	1.1380	0.6281
FlowDock	1.7074	0.6435	1.5671	0.5332	1.6087	0.4872	1.3229	0.4935
EHIGN	1.1973	0.7210	1.1506	0.7449	1.2741	0.6571	1.2649	0.6638
Boltz-2	1.2656	0.7652	1.2240	0.7409	1.1512	0.5768	1.1679	0.5936
Ours	**1.0391**	**0.8334**	**0.9245**	**0.8466**	**1.0683**	**0.6628**	**1.0590**	**0.6672**

To Reviewer #3

Reviewer #3, Question 1: *Some similar/related work is not mentioned and discussing similarities and differences would be beneficial to provide the reader with relevant context: https://arxiv.org/html/2505.19014v1, https://pubs.rsc.org/en/content/articlehtml/2024/ra/d3ra08650, potentially older work on QTAIM by Bader et al.*

Response: Thank you for the advice. We have added a discussion of similar work in the *Structural Preprocessing for Multi-Bond Interactions* section (lines 482-489) and the *Introduction* section (line 58). Specifically, prior work[13][14][15] has demonstrated that using electron cloud structure as a prior can provide binding information that atom-level molecular graphs cannot fully capture, enabling a more direct characterization of non-covalent interactions. However, such methods often compress and replace the original 3D electron cloud via pretrained hierarchical codebooks or electron-density topological descriptors based on critical points (BCP/NCP). It inevitably degrades the electron density's shape and intensity distribution in three-dimensional space. Moreover, because the representation is built on tokens or critical points, it often lacks explicit modeling of atom nodes and their covalent-bond topology in the molecular graph.

In addition, we have incorporated a citation to Bader et al.'s discussion in Atoms in Molecules[16] in the *3D Point Cloud Encoder for Non-Covalent Feature* section (lines 553-555) of the manuscript, together with the statement: "Consistent with the QTAIM[16] perspective that electron density features attractors associated with nuclei, clustering high-density regions yields centroids that tend to lie close to the corresponding atomic centers (i.e., near the true atomic positions)."

Reviewer #3, Question 2: *"E-CloudBind employs Gaussian sampling within adaptive envelopes (bottom) to generate a point cloud representation that is inherently rotation- and translation-invariant" → please explain more in detail. Do you mean rotation/translations to the entire structure? If so, the same argument holds for purely distance-based methods.*

Response: We generate an electron-cloud point set for the pocket by performing multivariate Gaussian sampling within an adaptive envelope centered at pocket atoms. The purpose is to approximate the electron-density distribution within the pocket region, and the resulting point cloud serves as the geometric input from which the downstream E-CloudBind can extract rotation- and translation-invariant features.

Specifically, we do not explicitly apply random rotation/translation augmentation to pockets during training. The invariance primarily stems from the design of the point-cloud encoder, 3D-GCN[17] (Fig. 2c in the manuscript), which performs kernel matching within local receptive fields based on relative direction vectors and local geometric relations rather than relying on absolute global Cartesian coordinates. Thus, E-CloudBind successfully encodes the invariant properties of the electron cloud through a purely distance-based design. The original paper[17] discusses the resulting shift/scale invariance in Sec. 3.2 (the explanatory paragraph following Eq. (5)) and summarizes the invariance properties in Sec. 3.3.

To empirically validate rotation and translation invariance, we performed a new rigid transformation experiment (now added to the *Ablation and Generalization Analysis* section, lines 305-311). We applied joint rotations (0°, 15°, 30°, …, 150°, 165°, 180°) and fixed translations to the input structures. As shown in Table 2 (or Fig. 4e of the revised manuscript), the MAE and Pearson correlation coefficient (PR) remain nearly unchanged across these transformations, indicating strong stability under rigid coordinate transformations. This empirically confirms that our E-CloudBind processes these point-cloud inputs in a strictly rotation- and translation-invariant manner. We have expanded the discussion in the revised manuscript to make this mechanism explicitly clear to the readers.

Reviewer #3, Question 3: *Additional details on how electron-density based features are assigned to individual atoms following k-means clustering would be useful.*

Response: Thank you for the advice. We have added more details in the *3D Point Cloud Encoder for Non-Covalent Feature* section (lines 552-563) regarding the k-means clustering procedure and on how point-cloud features are assigned to individual atoms. As an illustrative example, we describe the procedure using a batch containing two ligands, *A* and *B*. Let ligand *A* contain N_1 atoms and ligand and *B* contain N_2 atoms. Their associated electron-cloud point sets have different sizes, denoted as $P_A \in \mathbb{R}^{M1\times 3}$ and $P_B \in \mathbb{R}^{M2\times 3}$, where $M_1 \neq M_2$.

To assign point-cloud features to individual atoms and construct batch inputs, we perform K-means clustering on the electron-cloud points of each molecule separately, with the number of clusters set equal to the number of atoms in that molecule. Specifically, we use $K = N_1$ for ligand *A* and $K = N_2$ for ligand *B*. As a result, P_A is partitioned into N_1 clusters $\{\mathcal{C}_i^A\}_{i=1}^{N1}$, and P_B is partitioned into N_2 clusters $\{\mathcal{C}_j^B\}_{j=1}^{N2}$.

Since electron density is typically concentrated around atomic centers[16], each cluster center serves as a local spatial anchor. We explicitly assign each cluster center to its nearest atom by computing Euclidean distances between cluster centers and atomic

coordinates, recording the corresponding atom index. After the atom index is determined for each cluster, a fixed-size local point set is constructed by retaining the S points closest to the cluster center. This yields stackable inputs $X_A \in \mathbb{R}^{1\times N_1 \times S\times 3}$ and $X_B \in \mathbb{R}^{1\times N_2 \times S\times 3}$. During batch construction, the atom dimension is unfolded into the batch dimension, so that the input to the 3D-GCN has a shape of $(N_1 + N_2, S, 3)$. After the 3D-GCN, we obtain per-cluster embeddings of shape $(N_1 + N_2, D)$, which are then indexed and reshaped back to $\mathbb{R}^{N_1\times D}$ and $\mathbb{R}^{N_2\times D}$, yielding atom-wise electron features for each molecule.

Reviewer #3, Question 4: *"The specific running results are in the supplementary information." → Please point to the specific section/table in the supporting information.*

Response: Thank you for the advice. We have revised the sentence (lines 168-171) in the manuscript to: Descriptions of all the above methods and the corresponding results are provided in the Supplementary Information, specifically in the *Benchmarking Against State-of-the-Art Methods* section, Supplementary Table 4 and Supplementary Figure 1.

Reviewer #3, Question 5: *Please clarify whether the models you're choosing as a comparison (SIGN, PSICHIC, and DMFF) have been trained on the same data splits as your model.*

Response: We trained all baseline models using the same data splits as E-CloudBind and adopted the default model configurations and training hyperparameters reported in the original papers. All baselines were evaluated under a 10-fold cross-validation protocol on the merged PDBbind2013/2016/2019 dataset. The corresponding train/validation/test split CSV files have been uploaded to Figshare.

Reviewer #3, Question 6: *Please comment on the choice of dataset split (random/structural/temporal) and on the benefits/shortcomings of the chosen strategy.*

Response: Thank you for the advice. We have added a discussion in the supplementary material (*Performance Evaluation in a Cold Start Scenario* section) describing the advantages and limitations of the three data-splitting strategies: one warm start scheme (Random) and two cold start schemes (Scaffold split and Sequence identity), together with their corresponding references.

Random split is the most commonly used data-splitting strategy. Its advantages lie in its simplicity and in its ability to reflect training stability and average performance under an in-distribution setting. However, due to its randomness, homologous proteins or ligands with highly similar scaffolds may appear in both the training and test sets.

This results in test data that are highly similar to the training cases, leading to implicit information leakage, or dataset bias, and tends to overestimate the model's true generalization capability[18]. To this end, we constructed out-of-distribution (OOD) data splits from both the protein and ligand perspectives to ensure that the test involves previously unseen cases[19][20]. The sequence-identity split is based on full-length protein sequence similarity. We use NW-align to compute pairwise sequence identities between proteins and apply a 30% similarity threshold to prevent highly similar proteins from being split across different subsets. Under this split, proteins in the validation and test sets are considered sequence-dissimilar to those in the training set. Correspondingly, the scaffold split constructs an OOD setting based on ligand scaffold similarity. Ligand structural similarity is quantified using Morgan fingerprint counts together with a set of physicochemical descriptors. We then apply threshold-based partitioning to ensure that ligands in the validation and test sets are structurally distinct from those in the training set. Under this split, validation and test ligands are considered dissimilar to the training ligands at the structural level, enabling a more realistic assessment of the model's generalization performance on novel-scaffold ligands.

Reviewer #3, Question 7: *"[In order to] quantify robustness to crystallographic resolution, atomic coordinates were progressively perturbed to mimic structures solved at 1.9, 2.7 and 3.4 Å" → I understand you've added random coordinate noise, is this correct? If so, this would severely distort the underlying structures, even though also low-resolution structures from crystallographic or cryo-EM experiments would show (mostly) internally-constistent bond-length/angles etc. As an alternative experiment, I would suggest not adding coordinate noise, but rather stratifying the results on a hold-out test set by the respective resolutions of the experiments, i.e. what's the error on structures with resolution < 1.9 A, 1.9 A <= resolution < 2.7 A etc.*

Response: We sincerely apologize for the confusing phrasing in the original manuscript. The reviewer's suggested alternative experiment is, in fact, exactly what we performed. We did not add artificial random noise to the atomic coordinates to simulate resolution. Instead, the resolution-robustness analyses in Fig. 3b and Fig. 4j are conducted using the experimental resolution annotations provided in the PDBbind dataset, where the structures span a resolution range of 0.75-3.48 Å (with smaller values indicating higher resolution). In crystallography and cryo-EM, resolution characterizes the highest spatial frequency supported by the experimental data (the level of structural detail that can be reliably resolved)[21]. It is not equivalent to a fixed coordinate perturbation that can be directly converted into an atomic displacement. A high-resolution structure typically provides more reliable backbone and side-chain

conformations, as well as more confident placement of solvent molecules, ions, and ligand atoms[22]. In contrast, at lower resolutions, the backbone can often still be assigned, but the uncertainty in side-chain positioning and local interpretation increases substantially.

In the resolution-robustness experiment, we computed the absolute error (AE) metric for each complex in the test set as the y-axis value and used the experimentally determined resolution annotated as the x-axis value. Simple linear regression was fitted to all samples, and the fitted line and its confidence band were plotted. As shown in Fig. 3b, E-CloudBind exhibits a smaller slope in the fitted line, indicating that AE is less sensitive to resolution variations within the resolution range covered by the test set. In other words, the model maintains stable and low errors across structures of different resolutions.

Reviewer #3, Question 8: *In Figure 2a, you're mentioning frontier orbital calculations, though my understanding from the remainder of the paper is that you're not limiting your analysis to frontier orbitals, but are rather including the entire electron density. It would be good to clarify this.*

Response: In Fig. 2a, panels a1-a3 illustrate the workflow used to construct the ligand electron cloud for E-CloudBind. Specifically, panel a1 indicates that we first perform semiempirical electronic structure calculations using GFN2-xTB and export the results as a "molden.input" file. This file contains the atomic coordinates and the orbital energies, occupations, and coefficients required for electron-density reconstruction. We have clarified in the caption of Fig. 2a that the HOMO/LUMO shown in panel a1 are for visualization only, while the model input is the total electron density reconstructed from all occupied orbitals (lines 105-106). In panel a2, Multiwfn is used to compute the total electron density field ρ(r) from the xTB-exported "molden.input" file, evaluating the density on a three-dimensional grid from all occupied molecular orbitals. Finally, in panel a3, the density grid is subjected to threshold filtering to convert the volumetric field into a sparse point cloud suitable for downstream modeling.

Reviewer #3, Question 9: *Fig 3c: bar plots with vertical bars would be easier to read and would be in line with Fig 3d. Please also ensure same ordering of methods for ease of comparison across subplots.*

Response: Thank you for the advice. We have revised Fig. 3c to use bar plots with vertical bars and ensured a consistent ordering of the compared methods across all subplots.

Reviewer #3, Question 10: *The axis labelling in Figure 3e could be improved. It's not quite clear to me what you mean by "complexity-condited heat map" (i.e., how is ligand or protein complexity measured? - there are many ways to do this). What does "better complexity" mean? Higher or lower?*

Response: Thank you for this constructive advice. We apologize for the unclear presentation and the ambiguous statements in Fig. 3e, which may have led to this misunderstanding because "complexity" can be defined in multiple ways. We have revised Fig. 3e and added the discussion in the fourth paragraph of the *Robustness Analysis Across Heterogeneous Structural Quality* section (lines 194-202 and 233-245) to clearly define the complexity measures and the axis ordering. Protein complexity is quantified by Relative Contact Order (RCO)[23] and increases from left to right, whereas ligand complexity is quantified by the Bertz complexity index (BertzCT)[24] and increases from top to bottom.

In this figure, "complexity-conditioned" means that we organize protein-ligand pairs by protein complexity (RCO) and ligand complexity (BertzCT), and visualize the corresponding absolute error (AE) as a heat map. The term is used to indicate stratification along these two complexity axes and does not imply that higher or lower complexity is inherently "better". In the original caption, "(lighter = better)" referred to better predictive performance (lower AE), not to "better complexity". To avoid confusion, we removed the term "better complexity" and revised the caption (lines 194-202) to: "Fig. 3e visualizes per-pair absolute error (AE) as heat maps across methods, with columns ordered by increasing protein structural complexity (Relative Contact Order, RCO) and rows by increasing ligand structural complexity (Bertz Complexity Index, BertzCT). Each cell represents a protein-ligand pair, and lighter colors denote lower AE."

Reviewer #3, Question 11: *Figure 3d: the AE distribution of "Ours" does not seem to correspond to median error of 0.870.*

Response: Thank you for pointing this out, and we apologize for the confusion caused by an error in Fig. 3d. The reported median error (0.870) was incorrectly transcribed during manuscript preparation. In addition, due to an oversight in the plotting script, an additional square root was applied to AE when generating the distribution, while the axis and caption were labeled as absolute error (AE).

Fig. 3d is used to quantify, on the DAVIS dataset, the distribution of per-pair absolute error deviations relative to the corresponding method's mean absolute error. Our model achieves a median deviation of 0.280, which is lower than those of DMFF, PSICHIC, and SIGN by 0.456, 0.397, and 0.442, respectively. We have revised the corresponding

erroneous statement in the fourth paragraph of the *Robustness Analysis Across Heterogeneous Structural Quality* section (lines 233-237), and corrected Fig. 3d and the caption (lines 194-197) accordingly. This correction does not affect conclusions, as the AE deviation distribution still exhibits the lowest median deviation and the smallest dispersion compared with the other baselines.

For transparency and reproducibility, we provide the raw data used to generate the revised Fig. 3d in the supplementary files, and we have re-checked the manuscript to ensure that related figures and numerical results are accurate and consistently reported.

Reviewer #3, Question 12: *"p-value as high as 0.703" → please clearly state the statistical test you're using here.*

Response: Thank you for the advice. We have added an explanation of the p-value in the second paragraph of the *Robustness Analysis Across Heterogeneous Structural Quality* section (lines 207-212) of the revised manuscript. We used a parametric F-test in this analysis. The reported p-value corresponds to the standard t-test on the slope coefficient in an ordinary least squares linear regression, testing whether the slope is significantly different from zero. A larger p-value indicates that no statistically significant linear trend is observed between absolute error and resolution. In the linear regression analysis in Fig. 3b, E-CloudBind yields a higher p-value than the three baselines, indicating improved robustness to resolution variations.

Reviewer #3, Question 13: *"negligible reduction in Pearson of 0.004" → "negligible reduction in Pearson correlation coefficient..." would be more correct (same in other sentences)*

Response: Thank you very much for the wording correction. We have revised "Pearson" to "Pearson correlation coefficient" throughout the manuscript.

Reviewer #3, Question 14: *Figure 3 vs Figure 4 → why do you switch the metric from MAE to RMSE?; same from Figure 4b <> 4d.*

Response: Thank you for pointing this out. To ensure consistency and avoid confusion from using different metrics, we have revised the manuscript and figures to report MAE throughout, and the conclusions remain unchanged. We previously used MAE in Fig. 3 and Fig. 4d (now Fig. 4i of the revised manuscript). In Fig. 4b, we additionally report RMSE to show that, even under a metric that penalizes larger errors more strongly, the electron-density clouds obtained from xTB quantum calculations and our proposed van der Waals Gaussian sampler remain numerically close.

Reviewer #3, Question 15: *Figure 4: since you're discussing speed-ups: could you describe the computation cost/walltime requirements for computing electron densities/feature preparation/model training/inference time? GFN2-xTB is quite fast, particularly for small ligands.*

Response: Thank you for the advice. We have added a computational cost comparison in Fig. 4c and included the training time and inference speed for 10 folds in Supplementary Table 3. Further details and discussion can be found in the *Ablation and Generalization Analysis* section (lines 261-271) of the revised manuscript.

We first compare the time required for quantum-derived electron-density modeling and the proposed Gaussian-based modeling to convert a molecular structure into the final input format used by the E-CloudBind. For the xTB-based quantum preprocessing pipeline, GFN2-xTB is first run to generate a "molden.input" file containing the wavefunction information. Multiwfn is then used to compute the volumetric density grid and export it as a "density.cub" file. Finally, sparsification and clustering are performed to obtain the electron-cloud point representation used as the input. Using GFN2-xTB and Multiwfn, the preprocessing over the full PDBbind dataset of 19,545 ligands required 5.9 h and 8.2 h, respectively. The sparsification and clustering stages took 4.4 h and 6.4 h, respectively. For the proposed Gaussian-based modeling approach, the preprocessing requires only a one-time offline procedure. Multivariate Gaussian sampling is performed for each atom based on its van der Waals radius, reducing the total runtime substantially to 0.5 h.

In addition, we report the training time and inference speed. E-CloudBind contains 1.79×10^6 learnable parameters and uses a fixed point-cloud input shape. The electron clouds generated by the two preprocessing methods are identical in format during training and inference, ensuring consistent runtimes. Training on an NVIDIA A800 GPU takes an average of 15 min 52 s per epoch, and the model converges after approximately 271 epochs. The per-fold time distribution is provided in the Supplementary Table 3. For inference on an NVIDIA RTX 4090, a single batch with a batch size of 5 requires 1.1 GFLOPs and takes 1.7891 s.

Reviewer #3, Question 16: *Figure 4 shows that "the inexpensive Gaussian procedure matches the quantum-derived variant" - doesn't this contradict some of the other points you've made in the paper, i.e. that electron density is a useful description to assess protein-ligand binding affinity?*

Response: We apologize for any misunderstanding caused by our description of Fig. 4a in the manuscript. We have revised the corresponding statement (line 280-281) in the manuscript to: "the inexpensive Gaussian procedure achieves predictive

performance comparable to the quantum-derived modeling". Fig. 4a presents an ablation study showing that quantum-derived electron-density modeling and the proposed Gaussian-based modeling exhibit very similar performance across the three test sets. However, quantum-derived electron-density modeling is more time-consuming, as quantified in Fig. 4c of the revised manuscript. Therefore, the inexpensive Gaussian procedure provides a practical replacement that maintains predictive performance while greatly reducing computational cost. Moreover, the comparison against other baseline methods in Supplementary Table 4 shows that E-CloudBind, which incorporates electron-cloud density, consistently outperforms SMILES- or graph-only approaches. This supports that electron-cloud density provides an effective representation for protein-ligand binding affinity prediction.

Reviewer #3, Question 17: *Fig. 4d, MAE plot: bar plots coming from the top are very confusing and I've never seen one.*

Response: Thank you for the advice. The top-down bar plots are uncommon and can be confusing. A bottom-up bar format provides a more intuitive visualization and facilitates direct comparison across methods, making the performance advantage of E-CloudBind clearer. We have revised Fig. 4d (Fig. 4i of the revised manuscript) to use bottom-up bars for consistency.

Reviewer #3, Question 18: *Fig 5: color bars are confusing because the label "0 → 1" with the arrow pointing downwards implies that lighter green means 1, and darker green means 0, though the opposite is the case. Please also show the colors for all other atoms, including carbon atoms, for better comparability. Please clearly explain which attention score (from which part of the model) you're reporting.*

Response: We apologize for the confusion. We have revised the color bar in Fig. 5 to make the mapping unambiguous and to clearly indicate that lighter green corresponds to 0 whereas darker green corresponds to 1.

In addition, we have added an explanation of the source of the attention scores in the *Multi-Bond Feature Fusion for Binding Affinity Prediction* section (lines 664-668). The attention scores reported in Fig. 5 are taken from the Bias Correction Module in the output head (Supplementary Table 2, output layer). Concretely, given 256-d node features h for ligand and pocket atoms and 256-d cross-molecule edge features e, we compute an edge-wise score $w = h_{src} + h_{dst} + e$ and apply Linear(256→1) followed by PReLU and softmax to obtain edge-wise attention scores of shape $(E, 1)$, where $E = E_{lp} + E_{pl}$ is the total number of cross-molecule edges, with E_{lp} denoting ligand→pocket edges and E_{pl} denoting pocket→ligand edges. For visualization, we

aggregate edge-level attention to ligand atoms using scatter-add aggregation by summing the edge attention scores over edges that share the ligand atom as the source (ligand→pocket) or as the target (pocket→ligand), and then adding the two directional sums to obtain a per-atom importance vector in $\mathbb{R}^{N_{\text{ligand}}}$. Atoms with no incident cross-molecule edges receive a score of zero. Higher scores indicate a larger contribution of the corresponding ligand atoms to the bias correction and the final prediction.

Reviewer #3, Question 19: *"Four representative protein-ligand complexes were selected (PDBs: 1BCU, 3PXF, 5WMT, and 6DAR)". Please explain your motivation for choosing these specific complexes. Also, you are not citing papers for any of these PDBs. Please do so.*

Response: Thank you for this advice. We have clarified the motivation for selecting these specific complexes in the *Case Analysis and Large-Scale Screening* section (lines 343-347) and the caption of Fig. 5, and added citations for the PDBs. Specifically, to illustrate that E-CloudBind can recognize non-covalent interactions across proteins with different structural complexities, we quantified protein topological complexity using Relative Contact Order (RCO)[23] and present complexes in increasing order of protein complexity as 3PXF[25], 6DAR[26], 5WMT[27], and 1BCU[28], with additional cases are provided in Supplementary Figure 4. In addition, we have added citations for the target proteins 4OON[29], 7RFS[30], and 4MAN[31] used in the high-throughput screening experiments.

Reviewer #3, Question 20: *Question on virtual screening: "The top 5% of candidates, ranked by the model's predicted affinity scores, were subjected to molecular docking for validation." Do I understand correctly that since the model separately aggregates protein and ligand sub-graphs, the relative orientation of the two is not taken into account, and so (e.g. for virtual screening), you don't need the full complex geometry, but just a conformation for the ligand and the protein separately? If so, one could argue that for structure-based approaches, you want to negatively score wrongly oriented poses - or is the argument that the network implicitly reasons over orientation?*

Response: E-CloudBind do not require the full complex and only rely on the individual conformations of the protein and the ligand. The point-cloud features extracted by the 3D-GCN[17] from the electron cloud are invariant to rigid transformations. This means that applying global rotation or translation to the electron-cloud points yields the same features after processing, as described in our response to *Reviewer #3, Question 2*, and supported by the rotational-invariance experiment in Fig. 4d of the revised manuscript. As a result, the model does not rely on the absolute orientation of the protein and ligand

while being able to reason about their relative orientation through its transformation-invariant feature.

***Reviewer #3, Question 21:** You've shown that your model is resistant to coordinate noise, and I'd be curious to see a negative control: At which point does the model break down? Clearly if you distort the structures too much, you wouldn't want your model to output a positive binding score, because it would be based on entirely incorrect geometries that would never bind. E.g. rotate the ligand in a non-binding pose (that would depend on the answers to the previous question), distort the ligand which would mean large internal strain, ...*

Response: The negative control you suggested is highly constructive. When the input geometry is excessively distorted and departs from physically plausible conformations, i.e., under non-rigid perturbations, the model should no longer produce a positive binding signal. Conversely, as discussed in our responses to *Reviewer #3, Question 2 and 20*, E-CloudBind implicitly reasons over orientation through a transformation-invariant feature. Therefore, for inference inputs that differ from the training structure only by geometry-preserving rigid transformations such as rotation and translation, the predicted binding score should remain unchanged. Accordingly, we designed a new negative control experiment (now added as Fig. 4d-g and detailed in the *Ablation and Generalization Analysis* section, lines 305-321) to establish the model's breaking point under (i) rigid transformations (rotations/translations that preserve geometry) and (ii) non-rigid distortions (deformations that disrupt the local topology and introduce unphysical internal strain).

For rigid transformations, E-CloudBind is expected to be stable because it uses an invariant point-cloud encoder, 3D-GCN (Fig. 2c). In particular, 3D-GCN performs convolution within local receptive fields using relative direction vectors and local geometric relations rather than global absolute coordinates, which remains stable under rigid coordinate changes. The derivation of this invariance is discussed in the original paper in Sec. 3.2 (*Deformable Kernels for 3D Graph Convolution* section), in the explanatory paragraph following Eq. (5). To empirically verify this in our task, we kept model parameters and the evaluation pipeline identical and jointly rotated the ligand and pocket electron-cloud point sets around the z-axis by 0° (original), 15°, 30°, …, 135°, 150°, 165°, and 180°, together with a fixed translation. As shown in Table 2 (or Fig. 4e of the revised manuscript), the metrics remain nearly unchanged across rotations, with deviations from the 0° baseline of only MAE 0.00408 ± 0.00240 and Pearson correlation coefficient (PR) 0.00094 ± 0.00092, indicating strong stability under rigid coordinate transforms.

For non-rigid transformations, we construct corrupted inputs with a method that first perturbs the ligand's structure and then disrupts the electron-cloud geometry in the affected regions. Specifically, we randomly perturb a fraction r of ligand edges to emulate internal strain and local conformational changes induced by non-rigid deformation. This procedure identifies a set of ligand atoms that are influenced by the perturbation. We then apply geometric corruption only within the electron-cloud clusters associated with these atoms. For each affected cluster, we randomly select a fraction r of points and replace their 3D coordinates with random samples. The sampling is not performed over the entire molecular space but is constrained by the bounding box of each affected cluster. We expand the box around its center by approximately 3-4 times and sample new coordinates within this enlarged local region. After this procedure, the local density distribution and geometric shape are explicitly altered, providing the model with an electron cloud whose local geometry is distorted. As reported in Table 3 (or Fig. 4g of the revised manuscript), we evaluate multiple corruption ratios r ranging from 0% up to 90%. We observe a monotonic degradation in performance as r increases, with higher MAE and lower Pearson correlation coefficient (PR). Predictions become unreliable under medium to high corruption levels. These results indicate that the model's discriminative capability relies on preserving correct local geometric patterns of the electron cloud. Mild corruption can still preserve part of the informative signal due to the structural properties of the point cloud, whereas substantial geometric disruption prevents the model from extracting reliable cues and degrades predictive performance.

Table 2. Rigid-Transform Invariance to Rotation and Translation.

Angle	Test2013 MAE	Test2013 PR	Test2016 MAE	Test2016 PR	Test2019 MAE	Test2019 PR
0 °	0.9973	0.8444	0.8938	0.8584	1.0700	0.6614
15°	0.9893	0.8463	0.8895	0.8590	1.0765	0.6590
30°	0.9904	0.8456	0.8901	0.8587	1.0766	0.6584
45°	0.9899	0.8455	0.8908	0.8584	1.0756	0.6590
60°	0.9928	0.8448	0.8917	0.8587	1.0722	0.6606
75°	0.9964	0.8444	0.8953	0.8581	1.0704	0.6612
90°	0.9961	0.8440	0.8917	0.8585	1.0731	0.6604
105°	0.9902	0.8455	0.8907	0.8588	1.0767	0.6589
120°	0.9899	0.8454	0.8899	0.8585	1.0772	0.6582
135°	0.9902	0.8453	0.8906	0.8584	1.0757	0.6590
150°	0.9916	0.8451	0.8917	0.8586	1.0722	0.6607
165°	0.9997	0.8435	0.8946	0.8579	1.0698	0.6614
180°	0.9973	0.8444	0.8938	0.8584	1.0700	0.6614

Table 3. Model performance under varying non-rigid geometric corruption ratios.

Ratio	Test2013 MAE	Test2013 PR	Test2016 MAE	Test2016 PR	Test2019 MAE	Test2019 PR
0.00	1.0032	0.8424	0.8927	0.8583	1.0699	0.6615
0.05	0.9943	0.8466	0.9574	0.8369	1.1299	0.6259
0.10	1.1611	0.7884	1.1145	0.7564	1.2193	0.5766
0.15	1.3821	0.6768	1.2681	0.6878	1.3674	0.5108
0.20	1.4063	0.6719	1.4702	0.6072	1.5579	0.4262
0.25	2.0813	0.2769	1.7389	0.5221	1.7737	0.3703
0.30	2.0935	0.4849	2.1341	0.4370	2.0811	0.3014
0.35	2.3935	0.4105	2.3600	0.3910	2.3433	0.2836
0.40	3.1007	0.2717	2.9021	0.3539	2.7421	0.2620
0.45	3.5609	0.2689	3.5762	0.3338	3.2214	0.2424
0.50	3.7776	0.3547	4.1829	0.2651	3.6784	0.2409
0.55	5.2577	0.2583	4.7818	0.3095	4.2497	0.2279
0.60	5.6175	0.3935	5.7816	0.2920	4.9142	0.2231
0.65	6.9086	0.2378	6.4100	0.2926	5.4806	0.2168
0.70	7.1911	0.2568	7.1620	0.3025	6.3008	0.2074
0.75	8.3564	0.3097	8.1285	0.2969	7.0631	0.2125
0.80	9.6735	0.2542	9.3916	0.2832	7.9236	0.2110
0.85	10.1590	0.2464	10.2757	0.2568	8.7251	0.2088
0.90	11.7326	0.2871	11.4017	0.2881	9.6562	0.2162

Reviewer #3, Question 22: *Additional details on the network architecture (e.g. in a table) would be appreciated, i.e. number of message passing aggregations, specific atom/bond features etc. Which features from the electron density are actually going into the network?*

Response: Thank you for the advice. We have added architectural details of E-CloudBind to Supplementary Table 2 in the supplementary material, including the input and output dimensions, the purpose of each module, and explanations of the notation for both the 3D-GCN used for electron-cloud processing and the heterogeneous graph neural network used for molecular-graph modeling. The model inputs include the 3D coordinates of the electron cloud, the relative direction vectors and relative distances with respect to center point.

Reviewer #3, Question 23: *Could you motivate your choice of global max-pooling over the downsampled ligand and protein features? Doesn't this mean you only take into*

account one atom from the ligand and one atom from the protein or are you max-pooling over a different dimension?

Response: We do not select a single atom from the ligand or protein. Instead, max-pooling is performed over the set of electron-cloud points associated with each atom to obtain its per-atom embedding. The resulting embedding preserves information from all points within each atom, rather than collapsing information across different atoms. Concretely, the electron-cloud tensor fed into the 3D-GCN is constructed by clustering points according to their corresponding atoms and stacking a fixed number of points per atom, yielding a three-dimensional structure of shape $(B \times N_{\text{atom}}, N_{\text{point}}, 3)$, where the atom dimension is flattened into the batch dimension (the motivation for this design is explained in our response to *Reviewer #3, Question 3*). After several layers of point-cloud convolution, we obtain feature tensors of shape $(B \times N_{\text{atom}}, N_{\text{point}}, 128)$. A downsampling step is then applied along the point dimension with a pooling rate r (e.g., (r=4)), producing $(B \times N_{\text{atom}}, N_{\text{point}} / r, 128)$. Global max-pooling is subsequently performed along the point dimension N_{point} / r, independently aggregating all point features associated with each atom into a single vector and yielding a per-atom representation of shape $(B \times N_{\text{atom}}, 128)$.

Reference

[1] Youle R J, Strasser A. The BCL-2 protein family: opposing activities that mediate cell death[J]. Nature Reviews Molecular Cell Biology, 2008, 9(1): 47-59.

[2] Birkinshaw R W, Gong J, Luo C S, et al. Structures of BCL-2 in complex with venetoclax reveal the molecular basis of resistance mutations[J]. Nature Communications, 2019, 10(1): 2385.

[3] Ertl P, Schuffenhauer A. Estimation of synthetic accessibility score of drug-like molecules based on molecular complexity and fragment contributions[J]. Journal of Cheminformatics, 2009, 1(1): 8.

[4] Coley C W, Rogers L, Green W H, et al. SCScore: synthetic complexity learned from a reaction corpus[J]. Journal of Chemical Information and Modeling, 2018, 58(2): 252-261.

[5] Voršilák M, Kolář M, Čmelo I, et al. SYBA: bayesian estimation of synthetic accessibility of organic compounds[J]. Journal of Cheminformatics, 2020, 12(1): 35.

[6] Kim J, Ryu S, Lee W, et al. SHARP: generating synthesizable molecules via fragment-based hierarchical action-space reinforcement learning for pareto optimization[J]. Journal of Chemical Information and Modeling, 2025, 65(21): 11601-11619.

[7] Karplus M, McCammon J A. Molecular dynamics simulations of biomolecules[J]. Nature Structural Biology, 2002, 9(9): 646-652.

[8] Lionta E, Spyrou G, K Vassilatis D, et al. Structure-based virtual screening for drug discovery: principles, applications and recent advances[J]. Current Topics in Medicinal Chemistry, 2014, 14(16): 1923-1938.

[9] Vasiliauskaité-Brooks I, Healey R D, Rochaix P, et al. Structure of a human intramembrane ceramidase explains enzymatic dysfunction found in leukodystrophy[J]. Nature Communications, 2018, 9(1): 5437.

[10] Durairaj J, Adeshina Y, Cao Z, et al. PLINDER: the protein-ligand interactions dataset and evaluation resource[C]//ICML'24 Workshop ML for Life and Material Science: From Theory to Industry Applications.

[11] Passaro S, Corso G, Wohlwend J, et al. Boltz-2: towards accurate and efficient binding affinity prediction[J]. BioRxiv, 2025.

[12] Morehead A, Cheng J. FlowDock: geometric flow matching for generative protein-ligand docking and affinity prediction[J]. Bioinformatics, 2025, 41(Supplement_1): i198-i206.

[13] Zhang O, Jin J, Lin H, et al. ECloudGen: access to broader chemical space for structure-based molecule generation[J]. BioRxiv, 2024.

[14] Lin H, Zhang O, Xu J, et al. Tokenizing electron cloud in protein-ligand interaction learning[J]. ArXiv Preprint ArXiv:2505.19014, 2025.

[15] Isert C, Atz K, Riniker S, et al. Exploring protein-ligand binding affinity prediction with electron density-based geometric deep learning[J]. RSC Advances, 2024, 14(7): 4492-4502.

[16] Bader R F W. Atoms in molecules[J]. Accounts of Chemical Research, 1985, 18(1): 9-15.

[17] Lin Z H, Huang S Y, Wang Y C F. Convolution in the cloud: learning deformable kernels in 3d graph convolution networks for point cloud analysis[C]//Proceedings of the IEEE/CVF Conference on Computer Vision and Pattern Recognition. 2020: 1800-1809.

[18] Luo Y, Liu Y, Peng J. Calibrated geometric deep learning improves kinase-drug binding predictions[J]. Nature Machine Intelligence, 2023, 5(12): 1390-1401.

[19] Li H, Peng J, Sidorov P, et al. Classical scoring functions for docking are unable to exploit large volumes of structural and interaction data[J]. Bioinformatics, 2019, 35(20): 3989-3995.

[20] Yang Z, Zhong W, Lv Q, et al. Interaction-based inductive bias in graph neural networks: enhancing protein-ligand binding affinity predictions from 3d structures[J]. IEEE Transactions on Pattern Analysis and Machine Intelligence, 2024, 46(12): 8191-8208.

[21] Wlodawer A, Minor W, Dauter Z, et al. Protein crystallography for non-crystallographers, or how to get the best (but not more) from published macromolecular structures[J]. The FEBS Journal, 2008, 275(1): 1-21.

[22] Jeliazkov J R, Robinson A C, García-Moreno E B, et al. Toward the computational design of protein crystals with improved resolution[J]. Biological Crystallography, 2019, 75(11): 1015-1027.

[23] Plaxco K W, Simons K T, Baker D. Contact order, transition state placement and the refolding rates of single domain proteins[J]. Journal of Molecular Biology, 1998, 277(4): 985-994.

[24] Özçelik R, de Ruiter S, Criscuolo E, et al. Chemical language modeling with structured state space sequence models[J]. Nature Communications, 2024, 15(1): 6176.

[25] Betzi S, Alam R, Martin M, et al. Discovery of a potential allosteric ligand binding site in CDK2[J]. ACS Chemical Biology, 2011, 6(5): 492-501.

[26] Wang F, Jeon K O, Salovich J M, et al. Discovery of potent 2-Aryl-6, 7-dihydro-5 H-pyrrolo [1, 2-a] imidazoles as WDR5-WIN-site inhibitors using fragment-based methods and structure-based design[J]. Journal of Medicinal Chemistry, 2018, 61(13): 5623-5642.

[27] Deakyne J S, Malecka K A, Messick T E, et al. Structural and functional basis for an EBNA1 hexameric ring in Epstein-Barr virus episome maintenance[J]. Journal of Virology, 2017, 91(19): 10.1128/jvi. 01046-17.

[28] Conti E, Rivetti C, Wonacott A, et al. X-ray and spectrophotometric studies of the binding of proflavin to the S1 specificity pocket of human α-thrombin[J]. FEBS Letters, 1998, 425(2): 229-233.

[29] Starr J, Brown M F, Aschenbrenner L, et al. Siderophore receptor-mediated uptake of lactivicin analogues in gram-negative bacteria[J]. Journal of Medicinal Chemistry, 2014, 57(9): 3845-3855.

[30] Owen D R, Allerton C M N, Anderson A S, et al. An oral SARS-CoV-2 Mpro inhibitor clinical candidate for the treatment of COVID-19[J]. Science, 2021, 374(6575): 1586-1593.

[31] Souers A J, Leverson J D, Boghaert E R, et al. ABT-199, a potent and selective BCL-2 inhibitor, achieves antitumor activity while sparing platelets[J]. Nature Medicine, 2013, 19(2): 202-208.
